# Thermostability of Well-Ordered HIV Spikes Correlates with the Elicitation of Autologous Tier 2 Neutralizing Antibodies

**DOI:** 10.1371/journal.ppat.1005767

**Published:** 2016-08-03

**Authors:** Yu Feng, Karen Tran, Shridhar Bale, Shailendra Kumar, Javier Guenaga, Richard Wilson, Natalia de Val, Heather Arendt, Joanne DeStefano, Andrew B. Ward, Richard T. Wyatt

**Affiliations:** 1 IAVI Neutralizing Center at TSRI, Department of Research and Development, International AIDS Vaccine Initiative, La Jolla, California, United States of America; 2 Department of Immunology and Microbial Science, The Scripps Research Institute, La Jolla, California, United States of America; 3 The Scripps CHAVI-ID, The Scripps Research Institute, La Jolla, California, United States of America; 4 Department of Integrative Structural and Computational Biology, The Scripps Research Institute, La Jolla, California, United States of America; 5 IAVI Design and Development Lab, Department of Research and Development, International AIDS Vaccine Initiative, Brooklyn, New York, United States of America; University of Zurich, SWITZERLAND

## Abstract

In the context of HIV vaccine design and development, HIV-1 spike mimetics displaying a range of stabilities were evaluated to determine whether more stable, well-ordered trimers would more efficiently elicit neutralizing antibodies. To begin, *in vitro* analysis of trimers derived from the cysteine-stabilized SOSIP platform or the uncleaved, covalently linked NFL platform were evaluated. These native-like trimers, derived from HIV subtypes A, B, and C, displayed a range of thermostabilities, and were “stress-tested” at varying temperatures as a prelude to in vivo immunogenicity. Analysis was performed both in the absence and in the presence of two different adjuvants. Since partial trimer degradation was detected at 37°C before or after formulation with adjuvant, we sought to remedy such an undesirable outcome. Cross-linking (fixing) of the well-ordered trimers with glutaraldehyde increased overall thermostability, maintenance of well-ordered trimer integrity without or with adjuvant, and increased resistance to solid phase-associated trimer unfolding. Immunization of unfixed and fixed well-ordered trimers into animals revealed that the elicited tier 2 autologous neutralizing activity correlated with overall trimer thermostability, or melting temperature (T_m_). Glutaraldehyde fixation also led to higher tier 2 autologous neutralization titers. These results link retention of trimer quaternary packing with elicitation of tier 2 autologous neutralizing activity, providing important insights for HIV-1 vaccine design.

## Author Summary

As the sole determinant exposed on the viral surface to the host B cells, development of native-like HIV-1 envelope glycoprotein (Env) functional spikes has been a major initial objective in HIV-1 vaccine design. As immunogens, these trimer mimetics should remain stable in a native-like conformation to preferentially present conserved neutralizing epitopes, as opposed to non-neutralizing epitopes, to better elicit neutralizing B cell responses and antibodies in vivo during the immune response. We assessed SOSIP or NFL trimers displaying a range of stabilities, including chemical fixation. We demonstrate that increased resistance to high temperature-induced unfolding correlated with enhanced elicitation of tier 2 autologous neutralizing antibodies that are capable of penetrating this well-shielded viral pathogen, an important consideration for HIV vaccine development.

## Introduction

Quaternary packing of the heavily glycosylated HIV-1 envelope glycoprotein (Env) functional spike presents formidable obstacles for the elicitation of neutralizing antibodies to this viral surface unit. These obstacles, which have been defined over the past 20 years, include conserved epitope occlusion at the receptor CD4 binding site (CD4bs), receptor-induced formation of co-receptor binding sites, adaptable and extensive glycan shielding, spike subunit dissociation, and the umbrella shape of the trimer itself that restricts B-cell receptor access to underside regions of the spike [1–4]. These evolved elements render HIV relatively resistant to most host generated antibodies. In addition, the limited number of functional spikes on the virus or pseudo-typed virus like particles (VLPs) makes them less useful as immunogens, reducing overall Env immunogenicity and limiting the benefit of bivalent antibody avidity to the viral spike.

An alternative approach to VLPs is to generate well-ordered, soluble spike mimetics such as the recently described SOSIP or NFL trimers. These trimers consist of three protomers containing the gp120 binding domain covalently coupled to the gp41 ectodomain by two different strategies, which results in the generation of soluble, “native-like” HIV-1 spike mimetics [4–9]. With these trimers in hand, elicitation of tier 2 autologous neutralizing antibodies has been accomplished [10], leading to the hope that some of these antibody lineages can evolve into tier 2 heterologous neutralizing antibodies capable of neutralizing diverse strains. As immunogens, the well-ordered trimers should be stable for an extended period of time during the germinal center (GC) reaction in lymph nodes to elicit the proper B cell responses against the preferentially presented quaternary-dependent (i.e. an epitope created within each protomer by trimer context-dependent packing or across protomers) or angle-occluded, shielded and conserved neutralizing epitopes. We envision that potent adjuvants aid in the delivery of trimers to lymph nodes where they must maintain their native state, with limited conformational “breathing” at normal body temperature and amidst proteases, which may negatively impact stability, to selectively and continually present neutralizing determinants to B cells during the GC reaction. Since the high-resolution structure of the SOSIP native-like trimers is available [4, 8, 9], we and other groups are attempting to increase stability by structure-based design to potentially increase the maintenance of quaternary packing *in vivo* during the immunization and affinity maturation process to better elicit trimer-associated neutralizing antibodies. Alternatively, proteins can be stabilized through chemical cross-linking reagents. Several studies have explored the use of cross-linking reagents to stabilize soluble Env [11, 12] and membrane-expressed Env trimers [13, 14].

**Fig 1:**
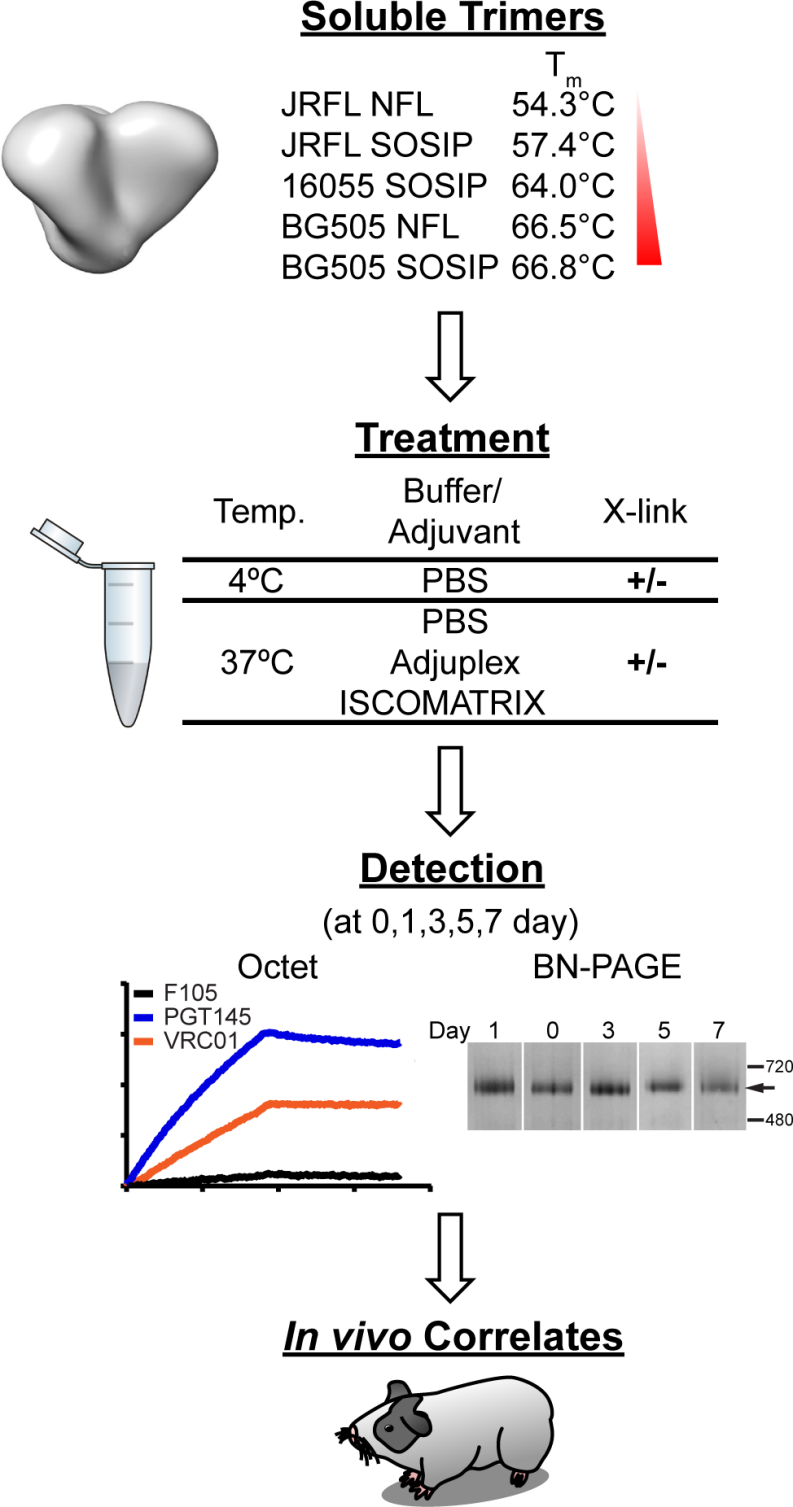
Schematic of stability test for well-ordered trimers. Representative soluble trimers from clade A, B and C in the NFL or SOSIP format were cross-linked with glutaraldehyde and/or treated under selected conditions (i.e. 4°C or 37°C and/or in adjuvants) for up to 7 days. Trimer stability was assessed by antibody binding profile and BN-PAGE using samples taken over the time course. Guinea pigs were immunized with selected trimers to access potential correlates between trimer stability and immunogenicity.

Therefore in this study, we assessed the solution stability of selected well-ordered trimers exhibiting a range of melting temperatures (T_m_), as measured by differential scanning calorimetry (DSC), under different conditions, including formulation with adjuvant at physiological temperature and following mild cross-linking with glutaraldehyde. A schematic of the overall experimental design is presented in Fig 1. We reasoned that trimers with a lower T_m_ would be more flexible and unstable, while trimers with a higher T_m_ would be more stable, better withstand environmental changes, and maintain proper native-like trimer formation in vivo. Glutaraldehyde cross-linking of selected trimers substantially increased the T_m_ and solution stability without generating higher order oligomers or significantly altering the trimer antigenic profile. To investigate in vitro stability with in vivo immunogenicity, we immunized guinea pigs with selected trimers derived from subtypes A, B and C and, in selected cases, following intra-trimer chemical cross-linking. The elicitation of tier 2 autologous neutralizing antibodies correlated with trimer thermostability as determined by calorimetry. Chemical cross-linking of the well-ordered trimers, besides substantially increasing thermostablity, elicited the highest levels of autologous neutralizing activity, especially against BG505 virus, correlating increased spike stability with the elicitation of enhanced tier 2 autologous neutralizing activity. Increased trimer stability may be an important factor to improve vaccine-induced neutralizing response to HIV [15, 16].

## Results

### Assessment of trimer stability by binding coupled with BN-PAGE

**Fig 2:**
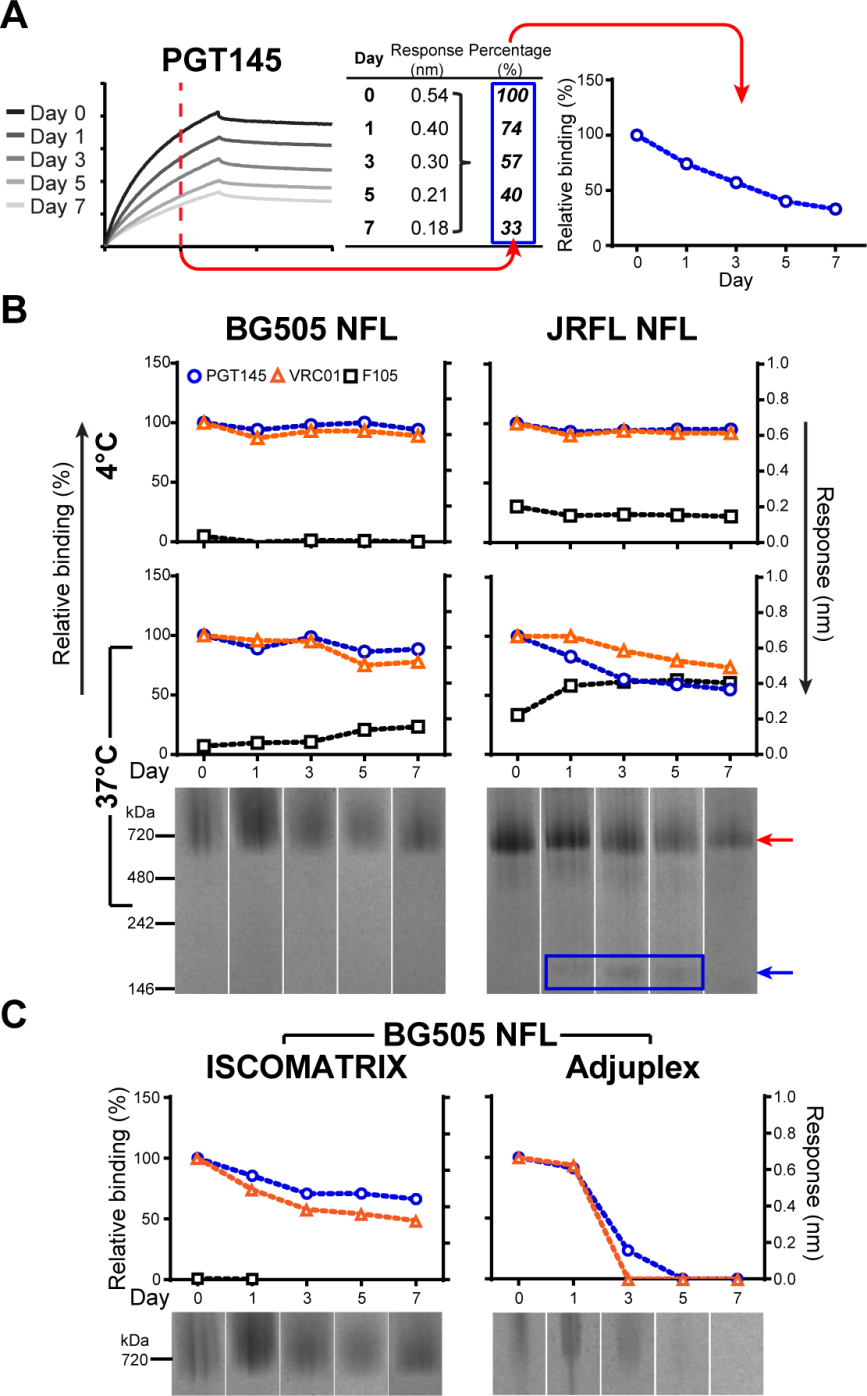
Stability of trimers in selected conditions. (A) Schematic showing how relative binding of the different mAbs to the trimers by BLI was determined. The binding response (nm) of the trimers from samples taken at day 1, 3, 5, and 7 was compared to the binding level at day 0 (100%) to calculate the relative binding (%) for each day. (B) Stability of BG505 NFL and JRFL NFL at 4°C and 37°C in PBS. Binding of VRC01, PGT145 and F105 was analyzed by BLI using anti-Fc sensors to capture the mAbs with the trimers as the analyte (top). Relative binding of VRC01 and PGT145 through the time course is plotted on left Y-axis. F105 binding level at each day was plotted on the right Y-axis (Response, nm). Stability of BG505 NFL and JRFL NFL at 37°C was also assessed by BN-PAGE shown below (trimer, red arrow; monomer, blue arrow). (C) Stability of BG505 NFL in ISCOMATRIX adjuvant and in Adjuplex at 37°C. The experiments were performed two times independently.

Although the T_m_ of recently characterized well-ordered trimers are 54°C or higher, there are concerns regarding trimer stability *in vivo* at 37°C and in the presence of potentially denaturing adjuvants. As such, we assembled a set of well-ordered trimer representatives from subtypes A (BG505), B (JRFL) and C (16055) displaying a range of thermostabilities, as determined by DSC, to further assess trimer stability under different conditions, including vaccine formulation for in vivo evaluation (see Fig 1). Initially here, we determined trimer stability at 4°C and 37°C in PBS pH 7.4 over the course of 7 days. Trimer stability was analyzed in two distinct ways. To help determine trimer quality and whether the trimers remained well-ordered, we assessed the antigenic profile of trimer samples using three characteristic monoclonal antibodies (mAbs) by biolayer interferometry (BLI). The broadly neutralizing antibody (bNAb) PGT145 was used to assess trimer cap integrity since PGT145 is a trimer-specific mAb that binds to the trimer apex in variable loop 2 (V2). The bNAb VRC01 binds to the CD4bs of gp120 and can recognize either the monomer or the trimeric forms of Env, and therefore can be used to measure total Env levels. The CD4bs mAb F105 recognizes monomer but not well-ordered trimers and can be used to assess open trimer, disordered trimer or trimer degradation into monomer. The mAbs were captured onto the sensors with the trimer as the analyte. A schematic of how relative binding of the mAbs is determined over the time course (day 0–7) is presented in Fig 2A. In parallel, we evaluated trimer integrity by Blue Native PAGE (BN-PAGE). By BN-PAGE, the trimer normally runs as a distinct single band at 690kD given its heavily glycosylated state. The band appears somewhat diffuse, or “fuzzy”, due to glycan heterogeneity with the nearly 100 N-linked glycosylation sites per intact trimer. As seen, when we incubated the trimers at 4°C over the course of 7 days in PBS, all trimers were relatively stable over this sampling period (Fig 2B upper panels and S1 Fig). PGT145 binding remained relatively constant, as did VRC01 binding, and F105 binding remained low.

Next, we assessed trimer stability at 37°C with varying results (Fig 2B lower panels). In general, the majority of the trimers remained intact over 7 days, but there was a hierarchy of stability that was associated with the respective trimer T_m_. The trimer with the highest T_m_, BG505 NFL (or BG505 SOSIP), remained relatively constant, as determined by the stability criteria. Recognition by the three mAbs was largely unchanged, with PGT145 and VRC01 remaining at 90% binding levels while F105 remained low, and the trimer remained intact as assessed by the native gels. In contrast, the trimer with the lowest T_m_, JRFL NFL, was increasingly recognized by F105 after 1 day at 37°C, while PGT145 and VRC01 binding decreased. A monomer band was detectable by BN-PAGE at this time point, consistent with ongoing trimer degradation. Meanwhile, JRFL SOSIP, which has an intermediate T_m_ value, reached a similar level of trimer degradation as JRFL NFL after 7 days, as measured by F105 and PGT145 binding. Similarly, 16055 SOSIP exhibited intermediate profiles by the selected stability criteria (S1 Fig).

### Trimer stability in selected adjuvants

We then assessed the stability of the trimers in two commonly used adjuvants at 37°C, ISCOMATRIX adjuvant (CSL) and Adjuplex (Advanced BioAdjuvants LLC; Sigma), which we have used in previous trimer immunogenicity studies, or similar adjuvants [17–20]. As seen in Figs 2C and S2, in ISCOMATRIX adjuvant at 37°C, we observed a gradual loss of both PGT145 and VRC01 binding to all the trimers over the 7 days even for the highly stable BG505 NFL/SOSIP, to levels approaching 50% of maximum. Consistent with these data, the trimer band signal also decreased on the BN-PAGE gel as assessed by visual inspection. However, F105 binding remained undetectable, perhaps indicating a different unfolding/degradation pathway than for JRFL NFL/SOSIP in ISCOMATRIX adjuvant at 37°C (S2 Fig). In contrast, both PGT145 and VRC01 binding decreased precipitously to undetectable levels by day 3 in Adjuplex, and the BN-PAGE trimer band became a smear by visual inspection by day 1 and no trimer band was detectable by day 5, consistent with overall trimer degradation for both BG505 NFL/SOSIP. Although BG505 SOSIP (S2 Fig) may appear to be slightly more stable than BG505 NFL (Fig 2C), it’s unclear whether a 5–10% difference in relative binding in vitro is significant or how that might translate relative to in vivo immunogenicity. The 16055 SOSIP and JRFL NFL and SOSIP trimers in ISCOMATRIX adjuvant or Adjuplex displayed similar stability profiles, unfolding relatively rapidly in either adjuvant (S2 Fig).

### Maintenance of order and homogeneity following homo-bifunctional cross-linking of trimers

**Fig 3:**
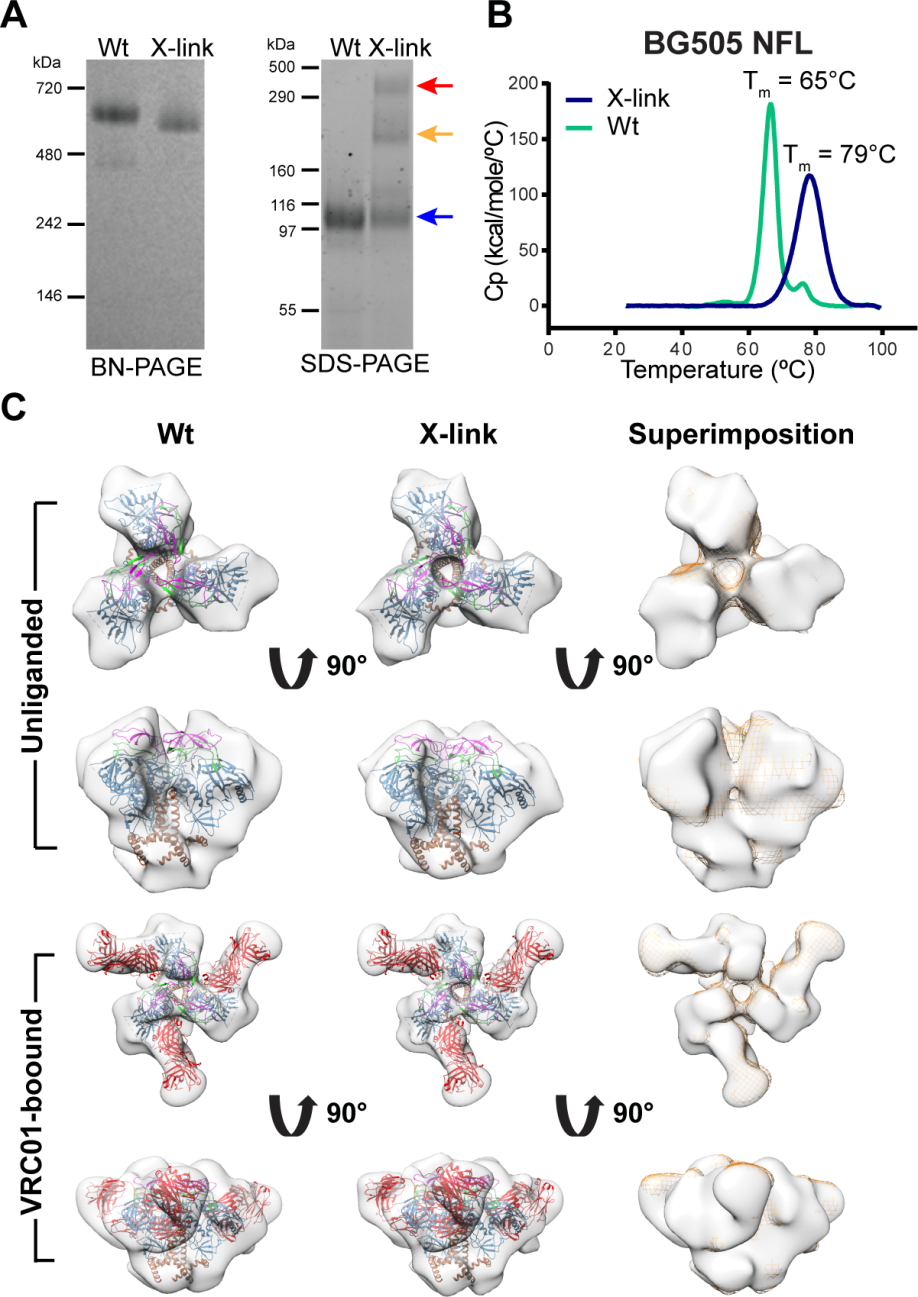
Characterization of well-ordered trimers before and after fixation with glutaraldehyde. (A) Comparison of trimers before (Wt) and after fixation (X-link) by BN- and SDS-PAGE. Bands corresponding to the trimer (red arrow), dimer (orange arrow), and monomer (blue arrow) are indicated. (B) DSC chromatograph of BG505 NFL before (Wt, blue) and after fixation (X-link, black). (C) EM 3D reconstructions comparing Wt (left) and X-link (middle) BG505 NFL trimer in the unliganded form (upper panel; Wt, EMD-8270; X-link, EMD-8271) or bound to VRC01 (lower panel; Wt, EMD-8269; X-link, EMD-8268). On the right, the Wt BG505 NFL EM density (solid gray) is superimposed on the X-link BG505 NFL trimer EM density (mesh orange). Top and side views are presented with the high-resolution cryo-EM BG505 SOSIP structure fitted within (PDB ID 3J5M; gp120 in blue, V1V2 in magenta, V3 in green, and gp41 in brown; VRC01, PDB ID 4S1Q, in red). The gel and DSC experiments were performed at least two times independently.

Due to the indications that the well-ordered trimers could unfold or degrade at 37°C in adjuvants, we sought to alter this outcome by chemical fixation. We selected the well-characterized homo-bifunctional cross-linker glutaraldehyde since we reasoned that the benefit of a bifunctional cross-linker might be to stabilize intra- or inter-protomer contacts that would enhance overall trimer stability. The optimal cross-linking procedure was determined to be 5 mM glutaraldehyde with 0.5 mg/ml protein for 5 min at room temperature. The reaction was subsequently quenched with 50 mM glycine, pH 7.5. We assessed the cross-linked (X-link or fixed) trimers by both BN- and SDS-PAGE analysis (Fig 3A). The X-link trimers migrated as a single band by BN-PAGE, displaying a slightly lower molecular weight compared to uncross-linked (Wt), consistent with a more compact conformation following fixation, but with no indication of cross trimer higher order cross-linking. Reducing SDS gel analysis revealed a single monomer band prior to fixation, but monomer, dimer and trimer bands following fixation, perhaps due to heterogeneous crosslinking. Interestingly, DSC revealed greatly increased mean T_m_ values with BG505 NFL/SOSIP approaching 80°C (plus 10 degrees), JRFL NFL at 65°C and JRFL SOSIP at 75°C (Figs 3B and S3). Broader DSC chromatogram profiles were detected for the less stable JRFL NFL and tighter profiles for the more stable BG505 NFL trimers.

We next assessed trimer conformation by negative-stain EM (Figs 3C and S4). The X-link trimers remained ~100% native-like and as discrete individual oligomers following fixation, with no detectable inter-trimer cross-linking consistent with the BN-PAGE data. 3D reconstructions of unliganded Wt and X-link BG505 NFL were obtained at 19 Å and 20 Å respectively, and superimposition of the two indicated comparable overall morphology (Fig 3C, upper panel). Further comparisons of the 3D reconstructions of Wt and X-link BG505 NFL in the VRC01 bound state at 21 Å and 20 Å, respectively, also showed little difference between the two (Fig 3C, lower panel).

**Fig 4:**
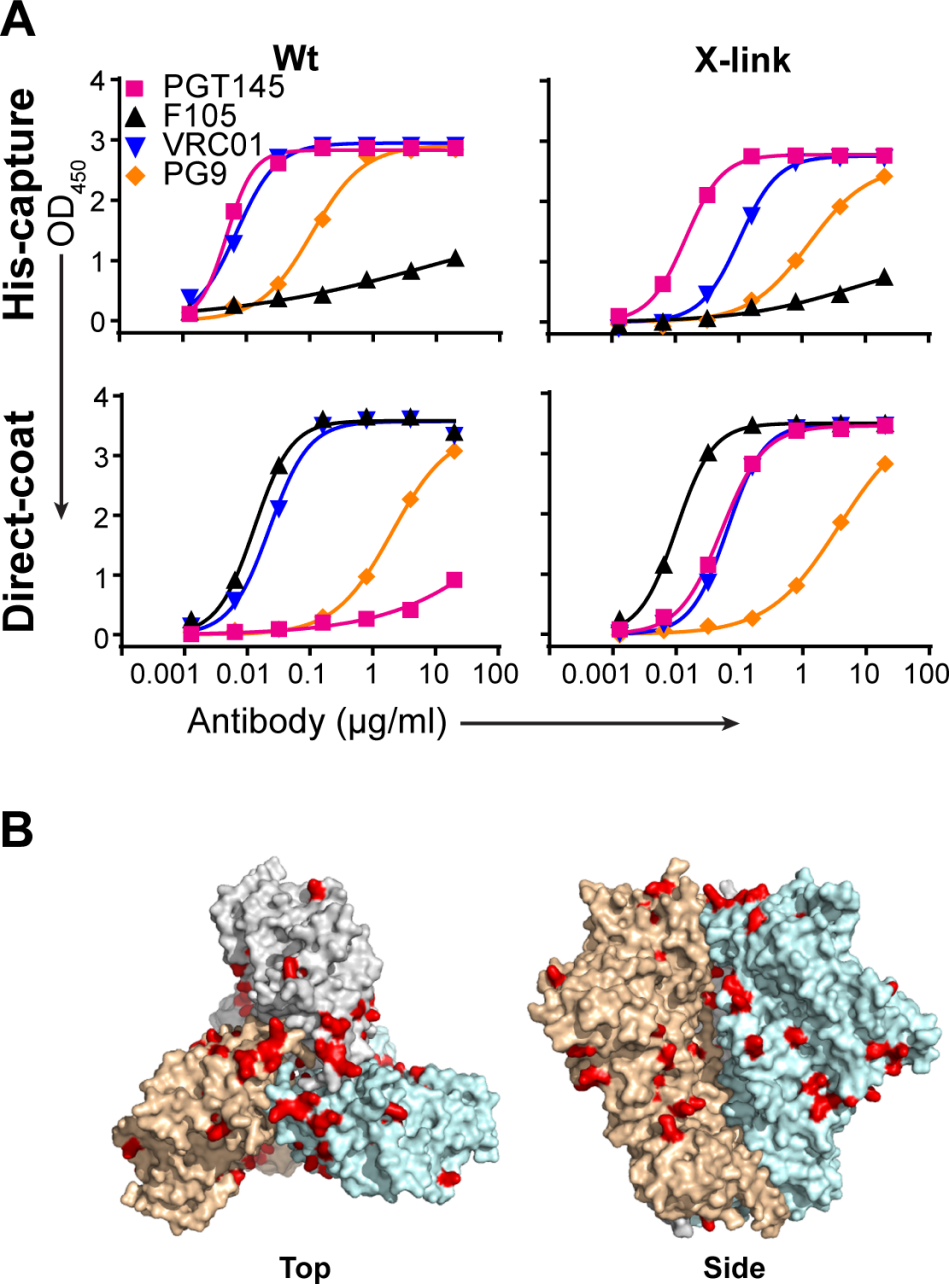
Antigenicity of Wt and X-link trimers. (A) Antigenic profile of Wt (left) and X-link (right) of JRFL NFL by ELISA using His-capture of the trimer (top) or with the trimer directly coated onto the plate (bottom). Binding of the trimer-preferring antibodies PGT145 (pink) and PG9 (orange), the broadly neutralizing CD4bs mAb, VRC01, and the non-neutralizing mAb, F105 are shown. The assay was performed two times independently. (B) Potential lysine residues (red) that can be cross-linked by glutaraldehyde are shown on the surface of BG505 SOSIP (PDB 4ZMJ) as an example.

We also characterized the antigenic profile of the trimers before and after cross-linking by ELISA and BLI (Figs 4A and S5). Using a His-capture ELISA format in which the trimers are captured by an anti-His mAb coated onto the plate, the trimers remain well-ordered as shown by high PGT145 recognition and low F105 (Fig 4, top left); however, if the trimers are directly coated onto the plate, then the inverse is seen, indicating the trimers unfold (Fig 4, bottom left). Analysis of the X-link trimers under the His-capture format showed that good PGT145 recognition with low F105 binding is preserved for X-link JRFL NFL (Fig 4A, top right), although some decrease in PGT145 binding of X-link BG505 NFL was observed. Interestingly, the X-link trimers retained quaternary-dependent antibody recognition after fixation in the direct-capture ELISA (Fig 4A, bottom right), suggesting that the solid-phase induced unfolding was partially remedied by fixation, as the trimers remained partially intact. That there was still F105 recognition following direct absorption onto the plate (Fig 4A, bottom right), indicated opening of some trimers in the population, likely from heterogeneous cross-linking of perhaps the numerous lysine (K) residues on the trimer surface (Fig 4B in red), which contain tertiary amines that are the most reactive moieties to interact with the aldehydes at neutral pH, although other functional groups can also be cross-linked by glutaraldehyde [21]. Thus, gluteraldehyde treatment has made the trimers relatively more stable, although a fraction of X-linked trimers still open up after adsorption to the ELISA plate. Without X-linking, all trimers would open up following adsorption.

Further binding assessments by BLI (S5 Fig) indicated comparable binding profiles between the Wt and X-link trimers with a couple exceptions. PGT145 binding to X-link BG505 NFL was lower compared to Wt, whereas binding remained similar between Wt and X-link JRFL NFL/SOSIP, as also shown by ELISA. This decrease in PGT145 recognition is consistent with the previously reported decrease seen with glutaraldehyde-fixed BG505 SOSIP [11, 22]. Although the PGT145 epitope in BG505 NFL appears to be affected by the fixation, the binding profiles of other trimer preferring bNAbs (i.e. PGT151, PG16, and VRC06) and all other antibodies tested remained relatively unchanged. Also, V3 antibody (i.e. 447-52D and 19b) binding to X-link JRFL SOSIP was reduced compared to Wt but no change was observed for X-link JRFL NFL.

**Fig 5:**
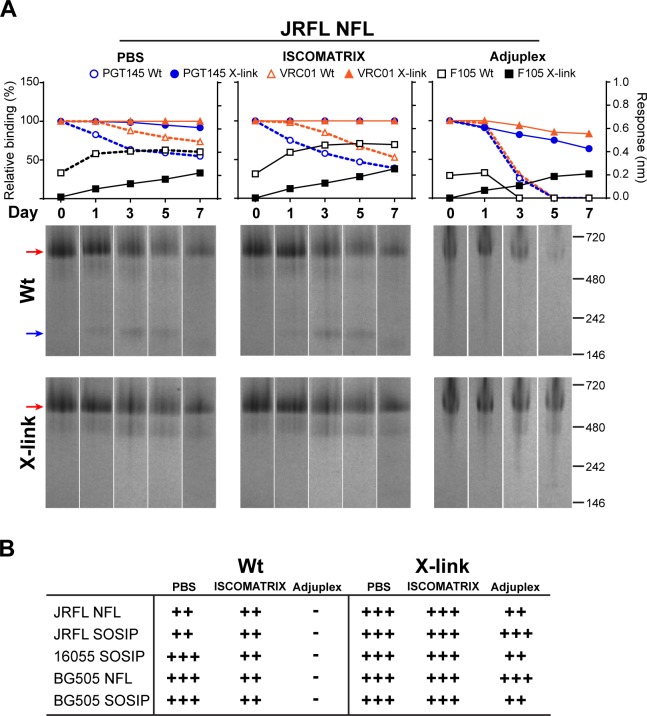
Stability of trimers after fixation in selected conditions. (A) Stability of JRFL NFL Wt (hollow symbol) and after glutaraldehyde fixation (X-link, solid symbol) in PBS, ISCOMATRIX adjuvant and Adjuplex at 37°C was detected by BLI (top) and BN-PAGE (bottom). The red arrow indicates the trimer and the blue arrow marks the monomer band on the gels. The experiments were performed two times independently. (B) Table summarizing the stability of NFL and SOSIP trimers at 37°C in selected conditions at day 7. Stable (+++) is defined as 80–100% relative binding of PGT145 and VRC01 with no increase in F105 binding, no monomer band and retains 80–100% trimer by BN-PAGE. Partially stable (++) is defined as 30–80% relative binding of PGT145 and VRC01 with an increase of F105 binding along with a visible monomer band and decreased trimer (30–80% remaining) by BN-PAGE. Unstable (–) is defined as < 30% relative binding of PGT145 and VRC01 with increased F105 binding coupled with a marked decrease (<30% remaining) of the trimer band by BN-PAGE.

Next, we assessed X-link trimer stability in adjuvant at 37°C (Figs 5 and S2) repeating the previous conditions with unfixed trimers as controls. Once again we observed that for the unfixed trimers, unfolding or degradation was detectable by both antibody binding and gel analysis and was associated with relative T_m_. In contrast, following fixation, all trimers appeared very stable by the binding criteria with high PGT145 and VRC01 recognition and lack of F105 recognition. Even the low T_m_ JRFL NFL trimers (Fig 5A) were extremely stable in ISCOMATRIX adjuvant and Adjuplex for 7 days at 37°C after cross-linking by both the binding criteria and BN-PAGE analysis. A summary of trimer stability in PBS or adjuvant pre- and post-cross-linking is presented in Fig 5B.

### Immunogenicity of well-ordered trimers of differing thermostabilities without and with fixation

**Fig 6:**
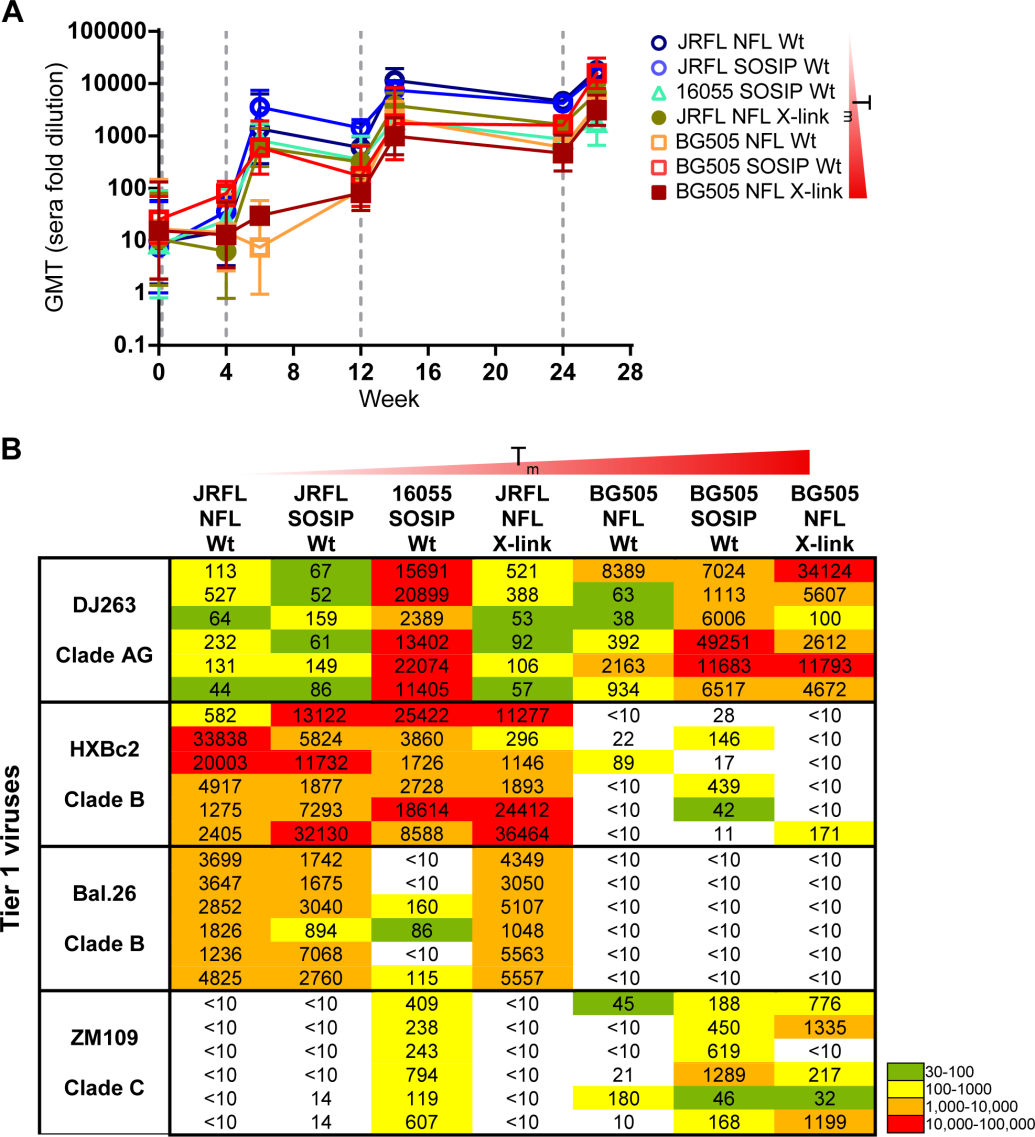
Immunogenicity of well-ordered trimers with or without fixation. Guinea pigs (*n* = 6) were immunized at week 0, 4, 12, and 24 with Wt JRFL, 16055, or BG505 SOSIP compared to JRFL or BG505 NFL with or without fixation (X-link). (A) Geometric mean IgG titers (GMT, ± SD; *n* = 6) are shown, as measured by His-capture ELISA to the autologous trimer immunogen (listed on the right by increasing T_m_). Sera were collected prior to inoculation (gray dash line) and at 2 weeks after. Assays were performed on two independent occasions with similar results. (B) Neutralizing ID_50_ titers (serum fold dilution factor) of sera collected two weeks after the fourth Env inoculation (wk 26) in guinea pigs (*n* = 6) against a panel of tier 1 viruses using the TZM-bl pseudovirus assay. The analysis was performed at multiple dilutions of each serum sample, and the data are representative of two independent experiments for most viruses.

Following the in vitro analysis, we tested the hypothesis that trimer stability, including chemical cross-linking, would increase the elicitation of neutralizing antibodies after vaccination. Accordingly, we immunized guinea pigs over a regimen of 0, 4, 12 and 24 weeks with Wt JRFL NFL, JRFL SOSIP, 16055 SOSIP, BG505 NFL and SOSIP trimers and X-link JRFL NFL and BG505 NFL trimers, for a total of 7 groups of 6 guinea pigs each. All trimers were formulated in ISCOMATRIX adjuvant since this adjuvant better maintained quaternary trimer integrity. Sera collected prior to immunization and two weeks post were assessed for binding antibodies to selected Env targets. Midpoint IgG binding titers were measured to each trimer immunogen captured in its near native state via its His_6_ C-terminal epitope tag by ELISA. Binding titers were detectable following the second immunization at 4 weeks, where they then waned to relatively low levels but were boosted following the third inoculation at 12 weeks. Titers again waned but increased beyond the week 14 titers after the fourth inoculation at week 24, exhibiting the typical “saw tooth” pattern for binding antibodies following Env immunization (Figs 6A and S6). In general, titers to the JRFL NFL and SOSIP immunogens were highest with BG505 NFL being lower and 16055 self-binding titers the lowest of the different trimer types. Interestingly, JRFL NFL, which has the lowest T_m_, elicited about 10 fold higher midpoint IgG binding titers than that of X-link BG505 NFL, which has the highest T_m_. Human mAb controls, such as the glycan-dependent bNAb, 2G12, indicated that the same amount of glycoprotein was captured on the ELISA plate for each trimer-type (S6 Fig).

Next, we assessed the capacity of the elicited serum antibodies to neutralize selected tier 1 and tier 2 HIV isolates. Inspecting tier 1 virus neutralization, the activity followed distinct patterns (Fig 6B). For the JRFL trimer-elicited serum, there was a strong capacity to neutralize the tier 1A clade B HXBc2 virus, which suggests there may be some CD4bs-targeted responses, since, under certain conditions, HXBc2 can be used as an indicator. This is because HXBc2 contains an insertion in the otherwise immunodominant N terminal V3 region [23] so that most vaccine-elicited sera do not cross react with the V3 loop, as long as the HXBc2 V3 or Env is not included in the immunogen. Such lack of V3 sensitivity is relatively rare for tier 1A viruses where neutralization has mapped predominantly to the variable regions, mostly V3 (e.g. BaL.26, MW965, SF162, etc.). Moreover, in the absence of primate CD4, we have shown that Env does not elicit CD4-induced antibodies in small animals [24], leaving the major sensitivity of HXBc2 to be at the CD4bs [17, 18, 25]. Beside HXBc2 neutralization, the JRFL trimer-elicited sera also potently neutralized the tier 1B clade B BaL.26 virus, but not the clade C ZM109 virus, likely due to V3 loop sequence differences. In contrast, the 16055 and BG505 trimers elicited relatively potent ZM109 neutralizing antibodies, but generally not to BaL.26. Of note, the most stable BG505 trimer antisera were very inefficient at neutralizing HXBc2, perhaps due to diminished exposure or immunogenicity at the conserved CD4bs on these highly ordered and stable trimers.

**Fig 7:**
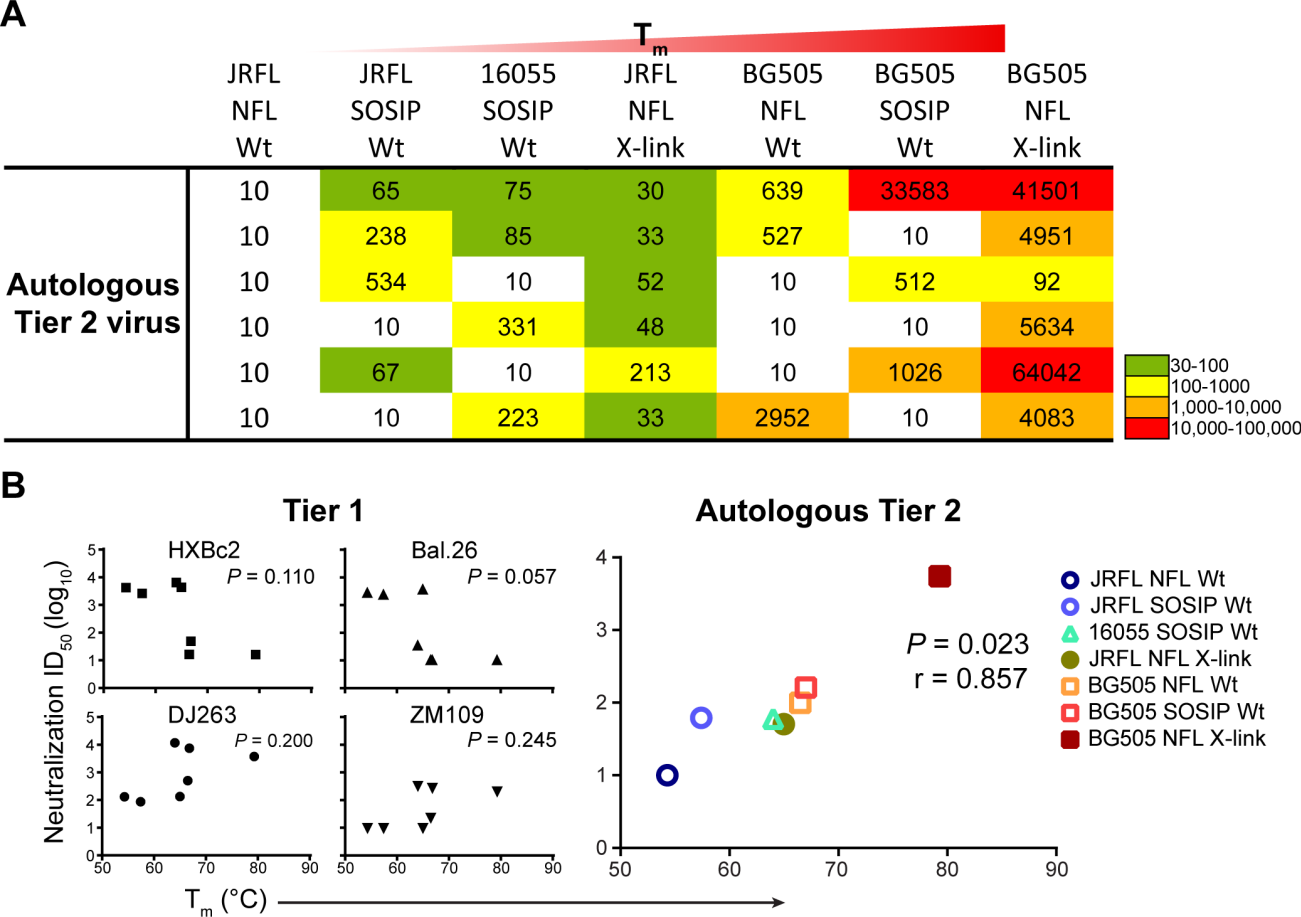
Autologous tier 2 neutralization correlates with trimer stability. (A) Autologous neutralization (ID_50_ titers, serum fold dilution factor) against tier 2 viruses elicited by JRFL, 16055, and BG505 well-ordered trimers, which are listed on top from left to right in increasing stability, as measured by T_m_. Neutralizing activity was measured in sera collected two weeks after the fourth Env (wk 26) inoculation in guinea pigs (*n* = 6) using the TZM-bl pseudovirus assay with multiple dilutions of each serum sample. The data are representative of at least three independent experiments. (B) Correlation analysis of stability and immunogenicity. Trimer T_m_ values were plotted against the serum neutralization titer (geometric mean) of tier 1 viruses and tier 2 autologous viruses. The P value of T_m_ vs tier 2 autologous neutralization (right) is 0.0238 by Spearman’s rank correlation analysis. No significant correlation was found between T_m_ and neutralization against tier 1 viruses.

Interestingly, the tier 2 autologous neutralization (Fig 7A) followed a pattern associated with the relative DSC-determined T_m_ in either the Wt or X-link trimer state. Wt JRFL NFL (54.3°C) did not elicit tier 2 autologous neutralization in any of 6 animals, whereas a few animals in each of the JRFL and 16055 SOSIP groups (57.4° and 64°C respectively) showed autologous neutralization; however, when the glutaraldehyde-cross-linked and more stable JRFL trimer (65°C) was used, all 6 animals exhibited modest JRFL neutralizing activity. For BG505, both Wt NFL and SOSIP trimers (66.5–66.8°C) elicited relatively potent tier 2 autologous neutralizing activities in 3 of the 6 animals from each group. Here too, trimer fixation of BG505 NFL (80°C) significantly increased the level of neutralizing activity with all 6 animals immunized with X-link BG505 NFL displaying detectable activity. Remarkably in two animals immunized with X-link BG505 NFL, the neutralizing ID_50_ values exceed 40,000 even though the binding titers to the trimer were relatively low. Although high autologous neutralizing titers were readily achieved in some cases, under the regimens performed in this study and with the selected heterologous isolates tested, there was no detectable tier 2 cross-neutralizing activity in most of the analyzed sera (S7 Fig). To confirm the relationship of increased stability with neutralization, we performed statistical analysis of both tier 1 and autologous tier 2 neutralizing activity and observed a correlation of DSC-determined T_m_ with tier 2 autologous neutralizing activity (p = 0.023 and r = 0.857), but no correlation of T_m_ relative to tier 1 neutralizing activity (Fig 7B). Recently, de Taeye et al. analyzed the autologous neutralization of BG505 and B41 SOSIP trimers displaying both increased stabilization and reduced exposure of V3 loop epitopes [16]. In that study, they did not detect a correlation with increased autologous neutralization. However, if we include those stability and neutralization data into our analysis here, the resulting merged data set resulted in an even stronger correlation of in vitro T_m_ with the elicitation of in vivo autologous neutralizing activity (p = 0.008; S8 Fig).

**Fig 8:**
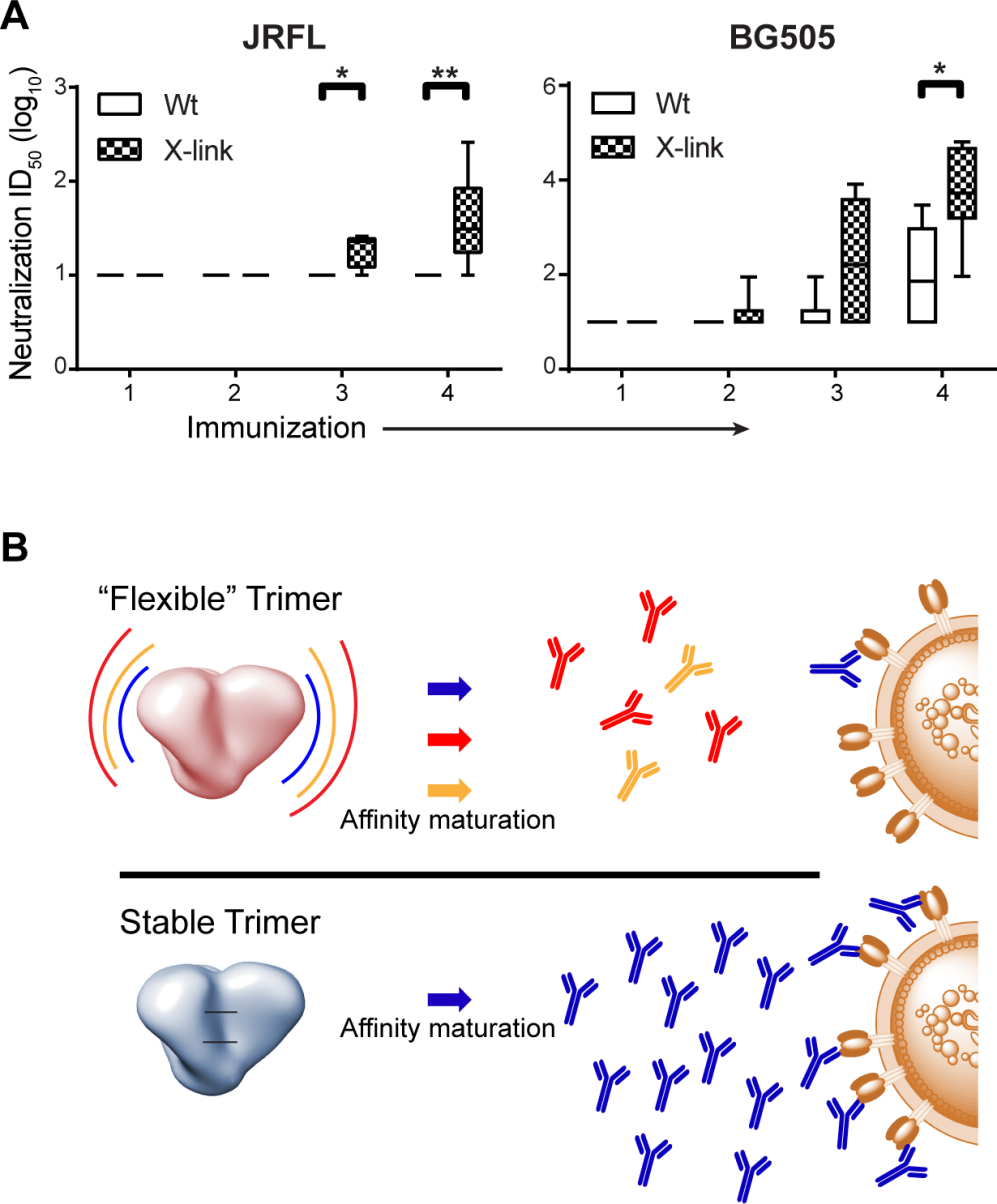
Increased trimer stability relates to improved elicitation of autologous neutralization. (A) Comparison of longitudinal tier 2 autologous neutralization elicited by Wt or X-link NFL trimers in guinea pigs (*n* = 6), as shown by box plots of neutralizing ID_50_ titers determined by the TZM-bl pseudovirus assay; horizontal line, median; box, interquartile range; whiskers, min/max. Left panel, JRFL autologous neutralization, *, P = 0.0152 after the third immunization, **, P = 0.0022 after the fourth immunization. Right panel, BG505 autologous neutralization, *, P = 0.0152 after the fourth immunization. Significance was evaluated by Mann-Whitney. The experiment was performed once using multiple dilutions of each serum sample. (B) Model depicting how native-like trimer stability, in particular when cross-linked (lower panel; black horizontal lines), presents limited conformations to the immune system during affinity maturation (block arrows) to generate a greater frequency of neutralizing antibodies (blue) that cross react with the functional spike on the virus. In contrast, less stable trimers (top panel) with lower T_m_ values (i.e. more flexible) may unfold more rapidly in adjuvant or in vivo, leading to the elicitation of more diverse B cell responses during the GC reaction, resulting in many different antibody specificities (red and yellow) that do not recognize the functional spike.

Since there was also no statistically significant increase of neutralizing activity against tier 1 viruses elicited by the X-link NFL trimers compared to Wt, this suggests that the improved autologous neutralization against tier 2 viruses likely comes from epitopes stabilized by the fixation rather than any potential adjuvant effect of glutaraldehyde on the immune system. To determine whether the more stable X-linked trimers could elicit autologous neutralizing antibodies earlier compared to Wt, we also assessed the autologous neutralization capacity of the sera after each immunization in a longitudinal manner (Fig 8A). For both JRFL and BG505 NFL, the X-link trimer groups displayed detectable autologous neutralizing activity before Wt and at the conclusion of the regimen. The increase in mean geometric neutralizing titers was statistically significant as determined by Mann Whitney.

### Analysis of the autologous neutralizing activity relative to the trimer and V3

To determine if the tier 2 autologous neutralization of the more stable X-link trimers was quaternary-dependent, we performed neutralization depletion assays as follows. Monomeric JRFL, 16055 and BG505 gp120 and ordered gp140 trimers were generated with the prototypic D368R point mutation in the CD4bs. 16055 SOSIP D368R did not trimerize and so was not available for analysis. Since these CD4bs “knockout” Envs do not bind CD4 (S10 Fig), we were able to pre-incubate them with neutralizing sera and leave them in solution during the TZM-bl entry assay. We could then determine if strain-matched monomeric or trimeric Env could adsorb or “deplete” neutralizing activity in the sera (Table 1 and S9 Fig). For the highly potent BG505 X-link or Wt trimer-elicited sera, all the tier 2 autologous neutralizing activity could be depleted with the gp120 monomer or gp140 trimer, indicating that the neutralizing antibodies were not strictly quaternary dependent. These data are in agreement with the recently published data using a similar analysis [10]. The results were more mixed for JRFL with some sera that could be inhibited with the monomer and/or trimer while a couple remained unaffected. For 16055, two of the neutralizing sera could be inhibited by addition of the monomer, consistent with the observation that most of the neutralizing activity was not quaternary-dependent.

**Table 1:** Mapping of autologous neutralization specificity by Env inhibition.

	Competitor		
Immunogen	gp120 D368R	NFL D368R	SOSIP D368R
**JRFL NFL X-linked**	4.4	–	
	Complete	Complete	
	–	–	
	Complete	Complete	
	4.9	3.2	
	3.4	3.7	
**BG505 NFL Wt**	Complete	Complete	
	Complete	Complete	
	Complete	Complete	
**BG505 NFL X-linked**	Complete	Complete	
	Complete	Complete	
	Complete	Complete	
	Complete	Complete	
	Complete	Complete	
	Complete	Complete	
**JRFL SOSIP Wt**	Complete		Complete
	6		Complete
	–		–
	Complete		Complete
**16055 SOSIP Wt**	Complete		
	4.5		
	26		
	Complete		
**BG505 SOSIP Wt**	Complete		Complete
	Complete		Complete
	Complete		Complete

Strain matched gp120 monomer or NFL/SOSIP trimer with the D368R mutation (to eliminate CD4 binding) were used as the competitor and pre-incubated with sera prior to addition of autologous virus in the TZM-bl neutralization assay. Fold reduction in neutralizing ID_50_ value with addition of competitor compared to mock are indicated unless complete or no (–) inhibition observed are as marked. Blank spaces, not determined.

We further determined whether the observed neutralizing activity could be mapped to the V3 loop region. While the V3 region is generally not a target of mAbs capable of neutralizing tier 2 isolates, it is an immunodominant region that often elicits neutralizing antibodies to tier 1 isolates in animals vaccinated with monomer or uncleaved gp140 [26]. Using the strain matched V3 peptide as the inhibitor, autologous neutralizing activity was largely unaffected (Table 2). Conversely, addition of the V3 peptide fully inhibited the majority of tier 1 cross neutralizing activity observed, while inhibition of ZM109 neutralization by addition of the BG505 V3 peptide was incomplete. Neutralizing activity to HXBc2, which is unmatched in the variable regions, was not markedly affected, as expected. These results are consistent with the generation of V3 reactive antibodies from well-ordered trimer immunized animals that can cross neutralize tier 1 isolates but do not account for the autologous tier 2 neutralization, as previously observed [10], although conformational V3 epitopes may not be detected by peptide inhibition.

**Table 2:** Analysis of neutralization activity by pre-incubation with V3 peptide.

	Tier 1A/B			Tier 2		
	Clade B		Clade C	Clade B	Clade C	Clade A
Immunogen	HXBc2	Bal.26	ZM109	JRFL	16055	BG505.T332N
JRFL NFL Wt	–	Complete				
	–	Complete				
	3.4	Complete				
	5.5	Complete				
	16.6	Complete				
	4.3	Complete				
JRFL NFL X-link	–	Complete		–		
	14.9	Complete		–		
	3.3	Complete		–		
	–	Complete		–		
	–	Complete		–		
	–	Complete		3.4		
JRFL SOSIP Wt	3.6	Complete		–		
	–	Complete		3.9		
	3.7	Complete		–		
	–	Complete				
	3.5	Complete		–		
	5	388				
16055 SOSIP Wt	–		Complete		–	
	–		Complete		–	
	–		Complete			
	–		Complete		–	
	4.3		Complete			
	4.4		Complete		–	
BG505 NFL Wt			Complete			–
						–
			Complete			
						–
BG505 NFL X-link			Complete			–
			Complete			–
			Complete			–
			Complete			–
			Complete			–
BG505 SOSIP Wt			Complete			–
			Complete			
			Complete			–
			20.3			
			Complete			–
			Complete			

Strain matched V3 peptide to the immunogen was pre-incubated with serum prior to addition of select virus in the TZM-bl neutralization assay. Fold reduction of neutralizing ID_50_ value with addition of V3 peptide compared to mock are indicated unless complete or no (–) inhibition observed. Blank spaces, not determined.

## Discussion

In principle, well-ordered trimers should be stable for an extended period of time during the GC reaction to elicit the proper B cell responses against quaternary-dependent or angle-occluded conserved epitopes accessible exclusively to tier 2 neutralizing antibodies. Selective and persistent presentation of neutralizing determinants by native-like and increasingly stabilized trimers to GC-maturing B cells is the driving concept behind this study (see schematic model in Fig 8B), and others, analyzing antibody responses elicited by these well-ordered Env trimers. In our study, we initially focused on 3 “sentinel” antibodies, in conjunction with gel analysis to evaluate trimer stability in vitro. These data, coupled with BN PAGE, selected EM, DSC analysis and antigenic profiling, provides definitive initial analysis, and one of the few of its kind, linking increased trimer stability in vitro to improvements in eliciting autologous tier 2 neutralizing antibody responses in vivo. Interestingly, within this more general theme, chemical cross-linking of the native-like trimers had the greatest effect on increasing trimer thermostability prior to vaccination as well as eliciting higher titer tier 2 autologous neutralizing activity following vaccination.

In terms of trimer stability in vitro, native gel analysis of the JRFL NFL trimer compared to the more stable BG505 NFL indicated that the JRFL trimers displayed a propensity to dissociate to monomers, whereas BG505 trimers appeared to degrade, perhaps due to low levels of protease present in the recombinant trimer preparation. Although chemical fixation does seem to overcome either of these unfolding pathways, the heterogeneity of the oligomeric subtypes detected by the SDS gel analysis following fixation indicated that not all trimers were similarly cross-linked, perhaps due to the disperse locations of the reactive K side chains on the solvent exposed surface of the trimers. In our study, chemical cross-linking of the trimers resulted in a large increase in thermostability for BG505 NFL, although not quite as large of an increase in the T_m_ as recently reported by the Sattentau group [11]. The resulting X-link BG505 NFL trimer DSC profile peak did broaden (i.e. greater T_1/2_), indicating some differences in the cross-linking process, but was more uniform and narrow as a single peak, signifying less sample heterogeneity overall, than the X-link BG505 SOSIP trimers. These differences may be due to slightly different cross-linking conditions or differences in the purification methods for the SOSIP trimers compared to the NFL trimers. That X-link JRFL NFL did not obtain as high as a T_m_ gain as X-link JRFL SOSIP may indicate that the slightly more stable JRFL SOSIP provided for a few more opportunities for cross-links to form.

That the chemically stabilized trimers of both JRFL and BG505 NFL origin became partially resistant to opening following ELISA plate adsorption and elicited enhanced tier 2 autologous neutralization relative to Wt trimers, suggested that some of this enhancement might be linked to quaternary-related neutralizing antibodies. However, even the potent BG505 tier 2 autologous neutralization observed could be inhibited completely by monomeric BG505 gp120, as could virtually all BG505-trimer elicited tier 2 autologous neutralization, whether generated from fixed or unfixed trimers. To note however, the BG505 monomer is somewhat unusual in that it is well-recognized by the usually quaternary-dependent bNAb, PG9, and does consistently elicit tier 2 autologous neutralizing antibodies in small animals as recently reported [10]. These properties may indicate that the conformation of the BG505 monomer is similar to its conformation on the trimer, relative to most other gp120 monomers. In contrast, for the JRFL X-link trimers, there were indications that some of the neutralizing antibodies may be quaternary-dependent. This apparent difference may be due to inherent relative trimer stabilities (JRFL less than BG505) and more heterogeneity, differential penetration of the fixative into the less stable JRFL trimers, or be a property of the more neutralization resistant JRFL virus. In the future, cloning of quaternary-specific mAbs from the JRFL immunized animals will confirm the indications from the adsorption analysis.

The lower JRFL autologous tier 2 titers may reflect less stability of the trimer, a more inherently neutralization-resistant virus, or exposure of less potent neutralizing determinants. Historically, the JRFL monomer does not elicit tier 2 autologous neutralizing antibodies [27], whereas the BG505 monomer does [10]. In fact, the relative rank order of the trimers by DSC-determined global stability was roughly associated with the ability to elicit tier 2 autologous neutralizing antibodies in vivo. The order from the lowest T_m_ to the highest, and elicitation of tier 2 autologous neutralization, being JRFL NFL<JRFL SOSIP<16055 SOSIP<JRFL NFL X-link<BG505 NFL/SOSIP<BG505 NFL X-link. This correlation was further strengthened with addition of data derived from the BG505 and B41 SOSIP trimers reported in the de Taeye et al. study [16], indicating the consistency of the results generated in different laboratories and in two different small animal models. Although not directly tested in the immunogenicity study presented here, given that X-link JRFL and BG505 NFL were able to more consistently elicit autologous neutralizing antibodies and at higher titers for X-link BG505 NFL compared to Wt, it would be expected then that X-link JRFL and BG505 SOSIP would also be able to more consistently elicit autologous neutralizing antibodies and at higher titers that Wt.

The most stable BG505 trimers were very poor at eliciting HXBc2 neutralization, considered an “open” tier 1A virus, which may reflect limited exposure of the occluded CD4bs on the most stable of the spikes assessed here. Alternatively, this might indicate that the cross reactivity at the CD4bs is low between BG505 (clade A) and HXBc2 (clade B). However, some of the BG505 elicited antisera could cross-neutralize HXBc2 and we have seen in another study in progress that more potent HXBc2 neutralization is indeed achievable with BG505 gp160 DNA, presumably due to incomplete cleavage resulting in open spikes akin to foldon trimers [28, 29]. These data suggest that strategies to better expose this site on the more stable trimers may be needed.

There was clear intra-clade bias for tier 1B neutralization and is likely due to differences in V3 sequences between clade B (GPGR and flanking residues) and the more similar clades A and C sequences (both with GPGQ at the tip). Not surprisingly, with the exception of the non-V3 matched HXBc2, most tier 1 elicited neutralizing activity mapped to either the clade B V3 (BaL.26) for JRFL or the clade C V3 (ZM109) for BG505 and 16055. Since V3 is slightly exposed on the JRFL SOSIP or NFL trimers [6, 7], elicitation of V3-directed tier 1 neutralizing activity was not unexpected. Although V3 exposure may be less with the BG505 trimers, some V3-directed neutralization was still elicited by these trimers, indicating that perhaps these trimers partially open in vivo. Of note, although X-link BG505 NFL elicited V3-directed antibodies, it also yielded the highest tier 2 autologous neutralization, indicating that such off target antibodies don’t necessarily preclude the efficient elicitation of tier 2 autologous neutralizing antibodies. Although efforts were made to eliminate or reduce V3 exposure in the BG505 SOSIP context, there was no significant improvement in autologous tier 2 neutralizing serum titers [16], consistent with our observation that V3 exposure does not readily affect the elicitation of autologous tier 2 neutralizing antibodies.

Glutaraldehyde has been safely used clinically with surgical transplants [30–32] and in other vaccines, including pertussis [33] and various allergy vaccines [34, 35]. While mostly used as a biocide to inactivate bacterial toxins and viruses, increasing antigen stability, as observed here, may serve as another utility in vaccine development. Alternatively, the enhanced immunogenicity detected for the high T_m_ glutaraldehyde-cross-linked trimers may also be influenced by an immune-potentiating adjuvant effect following the glutaraldehyde fixation process. The data presented here associating trimer stability with more efficient elicitation of autologous tier 2 neutralization in vivo portends favorably for emerging structure-based trimer designs possessing increased levels of quaternary stability [15, 16, 36]. These more targeted efforts may equal the benefits reported here regarding chemical fixation, and whether glutaraldehyde or other cross-linking reagents may add to this approach also warrants further study.

Ultimately of course, the long-term goal for a broadly effective HIV vaccine is to elicit antibodies capable of neutralizing diverse clinical isolates, which we have not achieved in this study. However, the design of this study was not directed toward that more challenging objective and was not the question addressed here. It should be noted that no licensed anti-viral vaccine elicits cross-protective antibodies. Even for the highly successful HPV vaccine, it appears that strain-matched antibodies confer protection. What is achieved here is increased tier 2 autologous neutralization, which is associated with trimer stability. It should be noted also that consistent and high-titer tier 2 autologous neutralization was not accomplished until relatively recently after decades of HIV Env immunogenicity experiments [10]. The specificities of this response are yet to be fully defined, and it may be that progress toward heterologous breadth may benefit from both the optimization and definition of tier 2 autologous neutralization and more informed vaccination strategies. Whether autologous tier 2 neutralizing antibodies, which begin to penetrate the highly effective glycan shield, lie on the pathway to the elicitation of heterologous neutralizing antibodies to any element of the functional spike is of course an extremely important question, but await additional and emerging analysis. Additionally, expectations that the homologous and relatively short term trimer regimens, such as those performed here, would be expected to elicit tier 2 heterologous neutralization should be tempered with both the low frequency and extended duration associated with the elicitation of heterologous neutralization generated during natural infection [37, 38], which remains as the most consistent positive readout towards this challenging vaccine goal.

## Materials and Methods

### Expression and purification of soluble Env trimers

The Env NFL and SOSIP trimers were produced as previously described [6, 7]. Briefly, the soluble NFL and SOSIP Envs were isolated by affinity chromatography using GNL (Galanthus nivalis lectin-agarose; Vector Labs), followed by negative selection using the non-neutralizing mAbs, F105 or GE136, for antibody affinity chromatography, and purified by size exclusion chromatography (SEC) using Superdex 200 columns (GE Healthcare Life Sciences) to isolate the predominant trimeric fractions. Site-directed mutagenesis was used to generate the D368R mutation in selected Env sequences. Trimers with the 368D/R mutation for the neutralization depletion assay were purified by a similar method described above, except non-neutralizing mAb b6 was used for the negative selection step. The gp120s were purified by cOmplete His-Tag Purification Resin (Roche Life Science), followed by SEC.

### Trimer cross-linking

The optimal concentration of glutaraldehyde (ACROS Organics) for cross-linking was determined by titration of 1 mM, 5 mM and 10 mM glutaraldehyde into 0.5 mg/ml of gp120 monomer or NFL trimers in PBS. Recognition of the X-link Envs by antibodies directed toward variable regions (14e, PG9), the CD4bs (VRC01, F105, VRC03), the co-receptor binding site (17b±sCD4), the C1-C5 region (C11) and quaternary epitopes (PGT145, VRC06) was evaluated by ELISA. By assessing the minimal change in antibody recognition for these antibodies to either gp120 or NFL, 5 mM of glutaraldehyde was selected as the best condition for cross-linking. Accordingly, 0.5 mg/ml of trimer was fixed with 5 mM glutaraldehyde at room temperature for 5 min and then the reaction was quenched by excess 50 mM glycine, pH7.5. The X-link trimers were negatively selected by F105 antibody affinity chromatography and re-isolated by Superdex 200 size exclusion chromatography.

### Longitudinal stability analysis of well-ordered trimers

Trimeric Envs without or with cross-linking were incubated up to 7 days in selected conditions, such as PBS pH 7.4 at 4°C, PBS pH 7.4 at 37°C, and ISCOMATRIX adjuvant or Adjuplex at 37°C. Each Env (15 ug in 15 μl PBS) was mixed with 75 units of ISCOMATRIX or 4 μl of Adjuplex. An aliquot of each sample was taken on day 0, 1, 3, 5, and 7. Trimer stability under these conditions was assessed by biolayer light interferometry (BLI) with a mini-panel of antibodies and BN-PAGE. The BLI analysis was performed as follows. The mAbs PGT145, VRC01 and F105 were captured by Anti-Human IgG Fc Capture (AHC) Biosensors on an Octet Red instrument (ForteBio, Pall life Sciences). After the baseline was established in 1X Kinetics Buffer (ForteBio), the sensors were dipped into wells containing 200 nM of aliquoted Env samples. The binding response (nm) detected at 120 seconds was used to calculate relative binding as follows (see also Fig 2A). The binding response of PGT145 or VRC01 to the day 0 sample (untreated) established the 100% binding level in the assay. Longitudinal relative binding level (%) was derived from the quotient of the binding response of each sample divided by the 100% level. For F105, the binding response of each sample was reported directly because F105 recognized only disordered trimer and no 100% binding value could be established to both ordered and disordered trimer.

### ELISA

His-capture ELISA was performed as previously described [7]. In brief, ELISA plates coated with 2 μg/ml of mouse anti-His mAb (R&D Systems) were first blocked in 5% nonfat milk and then used to capture the His_6_-tagged trimers. Serially diluted mAbs or sera from vaccinated animals were added into wells, and following incubation and washing, the secondary antibodies of peroxidase-conjugated goat anti-human IgG or goat anti-guinea pig IgG were added to all wells. Following incubation and washing, the plates were developed with the 3,3’,5,5’-tetramethylbenzidine chromogenic substrate solution (Life Technologies). For direct-coat ELISA, trimers were added directly to the wells at 2 μg/ml and analyzed as above.

### Differential scanning calorimetry

The thermal melting (T_m_) of the trimers was determined using a Microcal VP-Capillary DSC (Malvern). Briefly, trimers were diluted in PBS pH 7.4 to 0.25 mg/ml and scanned at a rate of 1°C/min. Data collected were analyzed after buffer correction, normalization and baseline subtraction using the VP-Capillary DSC Automated data analysis software.

### Animal immunization

Guinea pigs (*n* = 6 per group) were immunized intramuscularly in the quadriceps at 0, 4, 12, and 24 weeks with 20 μg of Wt JRFL NFL, JRFL SOSIP, 16055 SOSIP, BG505 NFL, BG505 SOSIP, X-link JRFL NFL or X-link BG505 NFL trimers, for a total of 7 groups. All Env trimers were formulated with 75 units of ISCOMATRIX adjuvant according to the manufacturer’s instruction. Serum was collected 14 days after each inoculation to assess binding and neutralization titers.

### Ethics statement

The guinea pig study was carried out under subcontract at Covance (Denver, PA), a site approved by the Association for Assessment and Accreditation of Laboratory Animal Care (AAALAC). The Covance Institutional Animal Care and Use Committee (IACUC) approved the study protocol (#0138–14), which was designed and conducted in strict accordance with the recommendations of the NIH *Guide for the Care and Use of Laboratory Animals* and the Animal Welfare Act and under the principles of the 3Rs. All efforts were made to minimize discomfort related to the inoculations and blood collection.

### Neutralization assays

A standard TZM-bl-based neutralization assay was performed as previously described [19] using tier 1 heterologous and tier 2 autologous pseudoviruses from clades A/G, A, B and C to evaluate neutralizing capacity of the serum from the vaccinated guinea pigs. Of note, BG505.T332N virus was used since both BG505 NFL and SOSIP also have the T332N mutation to restore the N332 glycan, the main binding determinant of several bNAbs [39]. Neutralization titers are expressed as the serum dilution factor sufficient to inhibit virus infection by 50% (ID_50_). Mann-Whitney statistical analysis was used to evaluate neutralizing titers between antisera from Wt and X-link trimer immunized animals. Spearman’s Rank Correlation analysis of neutralizing titers and DSC-determined T_m_ was performed using Prism 6 software (GraphPad). Inhibition of neutralization using the soluble monomer, trimer, or V3 peptide was performed as previously described [10, 19]. Briefly, serially diluted serum was pre-incubated for 1 hour at 37°C with 10 μg/ml of strain matched gp120 or trimer possessing the 368D/R mutation or 30 μg/ml peptide. The 368D/R mutation eliminates gp120 or trimer binding to CD4 on the TZM-bl target cells in the neutralization assay so that the proteins can be left in during assessment of viral entry. The extent of depletion of neutralizing capacity was calculated by the fold reduction of the ID_50_ value compared to the value derived without addition of the depleting reagent.

### Electron microscopy sample preparation

The purified trimers unliganded and bound to VRC01 were analyzed by negative stain EM. A 3 μL aliquot containing ~0.01 mg/mL of the sample was applied for 15 s onto a carbon-coated 400 Cu mesh grid that had been glow discharged at 20 mA for 30 s, then negatively stained with 2% uranyl formate for 45 s. Data were collected using a FEI Tecnai Spirit electron microscope operating at 120 kV, with an electron dose of ~35.66 e^-^/Å^2^ and a magnification of 52,000 x that resulted in a pixel size of 2.05 Å at the specimen plane. Images were acquired with a Tietz 4k × 4k TemCam-F416 CMOS camera using a nominal defocus of 1000 nm and the Leginon package [40] at 10° tilt increments, up to 40°. The tilts provided additional particle orientations to improve the image reconstructions.

### Data processing and image reconstruction

Particles were picked automatically using DoG Picker and put into a particle stack using the Appion software package [41, 42]. Reference-free, two-dimensional (2D) class averages were calculated using particles binned by two via the iterative msa/mra Clustering 2D Alignment [43] and IMAGIC software systems [44] and sorted into classes. To analyze the quality of the trimers (closed, open or non-native like trimers), the reference free 2D class averages were examined by eye using the same metrics previously described [45].

An *ab initio* common lines model was calculated from reference-free 2D class averages in EMAN2 [46] imposing symmetry C3. This model was then refined against raw particles for an additional 25 cycles. EMAN [47] was used for all 3D reconstructions. The resolutions of the final models were determined using a Fourier Shell Correlation (FSC) cut-off of 0.5.

For the 3D reconstruction of X-link BG505 NFL, a number of 19384 particles were used. For the 3D volume of BG505 NFL without fixation, a number of 22037 particles were used. For the 3D volume of X-link BG505 NFL bound to VRC01, 10234 particles were used, and for Wt BG505 NFL bound to VRC01, 17231 particles were used.

EM maps have been deposited in the EMDB as follows: unliganded BG505 NFL Wt (EMD-8270) or X-link (EMD-8271) and VRC01-bound BG505 NFL Wt (EMD-8269) or X-link (EMD-8268).

### Model fitting into the EM densities

The crystal structure of BG505 SOSIP (4NCO), was manually fitted into the EM densities of the unliganded trimers, while the crystal structure of gp120 bound to VRC01 (4S1Q) was manually fitted into the EM densities of the VRC01 bound trimers. All were refined using the UCSF Chimera program [48].

## Supporting Information

S1 Fig Stability of trimers at 4°C and 37°C in PBS. Binding of trimers maintained at 4° or 37°C to VRC01, PGT145 and F105 was analyzed by BLI (top). VRC01 and PGT145 binding levels at day 1, 3, 5 and 7 were compared to their binding level at day 0 to calculate the percent of “Relative binding” (y-axis, left). F105 binding levels were plotted as the binding response (nm; Y axis, right) measured by BLI. Bottom, stability of BG505 and JRFL NFL trimers at 37°C as detected by BN-PAGE are shown below (red arrow, trimer; blue arrow, monomer). The experiments were performed two times independently.
(TIF)

S2 Fig Stability of trimers formulated in adjuvant at 37°C. Trimers formulated in ISCOMATRIX adjuvant (A) or Adjuplex (B) were maintained at 37°C and assessed for binding to VRC01, PGT145 and F105 by BLI and stability visualized by BN-PAGE (red arrow, trimer; blue arrow, monomer). The experiments were performed two times independently.
(TIF)

S3 Fig Thermostability of trimers determined by differential scanning calorimetry (DSC). (A) Thermal transition melting (T_m_) curves of the different trimers with corresponding values (B) are shown (see Methods). All data are representative of at least two independent experiments.
(TIF)

S4 Fig Negative Stain EM data of BG505 NFL before and after cross-linking by glutaraldehyde. (A) 2D class averages of Wt (left) and X-link BG505 NFL trimers in the unliganded (top) or VRC01-bound state (bottom). (B) Distribution of native closed, native open or non-native like trimers of the unliganded Wt or X-link BG505 NFL samples were determined by analysis of over 2000 individual particles (see Methods). (C) The resolutions of the EM reconstructions were calculated from the Fourier Shell Correlation (FSC) using a cut-off of 0.5. The final resolutions obtained for the different BG505 NFL trimers obtained are as follows: unliganded Wt, 19 Å (upper left, EMD-8270); unliganded X-link, 20 Å (upper right, EMD-8271); VRC01-bound Wt, 21 Å (lower left, EMD-8269); VRC01-bound X-link, 20 Å (lower right, EMD-8268).
(TIF)

S5 Fig Antibody recognition of BG505 NFL and JRFL NFL before and after cross-linking. Antibody binding profiles of Wt (dashed lines) or X-link (solid lines) BG505 (blue) and JRFL (red) NFL were assessed by BLI using anti-human Fc sensors to capture the mAb with the trimer in solution. The different classes of mAbs tested include: CD4 binding site directed bNAbs (CD4-Ig2, VRC01, HJ16), trimer preferring bNAbs (PGT145, PG16, VRC06, PGT151), glycan dependent bNAb 2G12, and V3 directed mAbs (447-52D and 19b). All data are representative of at least two independent experiments.
(TIF)

S6 Fig Validation of the target trimers used in ELISA for the serum binding titers. VRC01, F105 and PGT145 binding levels were used to determine the quality of each trimer immunogen captured by His_6_ C-terminal tag on the ELISA plates. 2G12 was used to confirm that the same amount of trimer was captured in each well of the ELISA plate.
(TIF)

S7 Fig Tier 2 neutralization. Neutralization capacity of week 26 sera from guinea pigs (*n* = 6) immunized with select trimers (listed on the left) at 0, 4, 12, 24 weeks against a panel of heterologous tier 2 viruses was determined by the TZM-bl neutralization assay. ID_50_ values are shown. No breadth was observed from the limited tier 2 panel assessed. SIV was used as a negative control.
(TIF)

S8 Fig Correlation of Tier 2 autologous neutralization and thermostability of the immunogens. The autologous neutralization ID_50_ and trimer T_m_ values from the rabbit study reported in de Taeye et al. (Cell, 2015) were plotted together with our data as presented in Fig 7B. The T_m_ values are as follows: our study (JRFL NFL Wt, 54.3°C; JRFL SOSIP Wt, 57.4°C; 16055 SOSIP Wt, 64.0°C; JRFL NFL X-link, 65.0°C; BG505 NFL Wt, 66.5°C; BG505 SOSIP Wt, 67.0°C; BG505 NFL X-link, 79.3°C) and de Taeye’s study (B41 SOSIP Wt, 58.6°C; AMC008 SOSIP Wt, 60.2°C; B41 SOSIP v4.1, 61.7°C; AMC008 SOSIP v4.2, 64.0°C; AMC008 SOSIP v4.1, 64.5°C; BG505 SOSIP Wt, 66.7°C; BG505 SOSIP v4.2, 69.3°C; BG505 SOSIP v4.1, 69.5°C). Spearman’s rank correlation analysis showed a statistically significant correlation between the autologous neutralization ID_50_ and trimer T_m_, P value is 0.008 and r value is 0.670.
(TIF)

S9 Fig Validation of neutralization assay for sera mapping. (A) Strain matched CD4bs “knockout” gp120 D368R monomer, NFL/SOSIP D368R trimers were preincubated with mAbs (listed above) prior to addition of pseudovirus (listed on the left) in the TZM-bl neutralization assay. VRC01 (CD4bs-directed) and 2G12 (glycan reactive) can bind monomer and trimer while PGT145 binds trimer. (B) Representative example of V3 peptide inhibition assay. Sera samples were preincubated with media (mock), V3 peptide, or scrambled peptide prior to addition of pseudovirus in the TZM-bl neutralization assay.
(TIF)

S10 Fig Antigenic profile of D368R variants for sera mapping. Antibody binding profiles of the D368R variants used in the sera mapping experiment were assessed by BLI using anti-human Fc sensors to capture the mAbs with the trimer variants in solution. Results are summarized in the figure with “+” for binding and “-” for no binding.
(TIF)
