# Supplementary figures and images for: Thermostability of Well-Ordered HIV Spikes Correlates with the Elicitation of Autologous Tier 2 Neutralizing Antibodies
Source: PLoS Pathog. 2016 Aug 3;12(8):e1005767. doi: 10.1371/journal.ppat.1005767 (PMC4972253; doi:10.1371/journal.ppat.1005767)

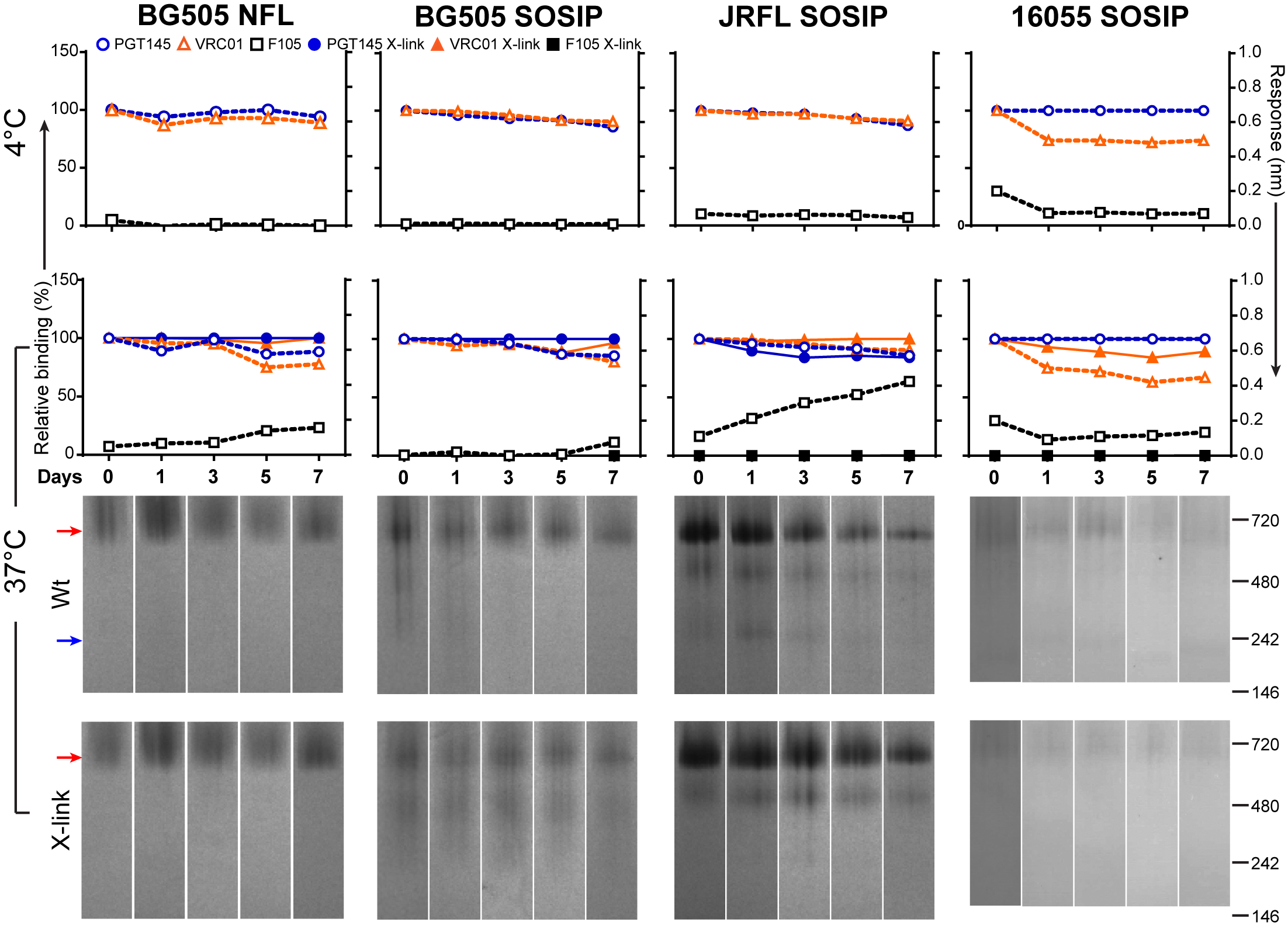

Supplement: S1 Fig — Binding of trimers maintained at 4° or 37°C to VRC01, PGT145 and F105 was analyzed by BLI (top). VRC01 and PGT145 binding levels at day 1, 3, 5 and 7 were compared to their binding level at day 0 to calculate the percent of “Relative binding” (y-axis, left). F105 binding levels were plotted as the binding response (nm; Y axis, right) measured by BLI. Bottom, stability of BG505 and JRFL NFL trimers at 37°C as detected by BN-PAGE are shown below (red arrow, trimer; blue arrow, monomer). The experiments were performed two times independently. (TIF) [file ppat.1005767.s001.tif]

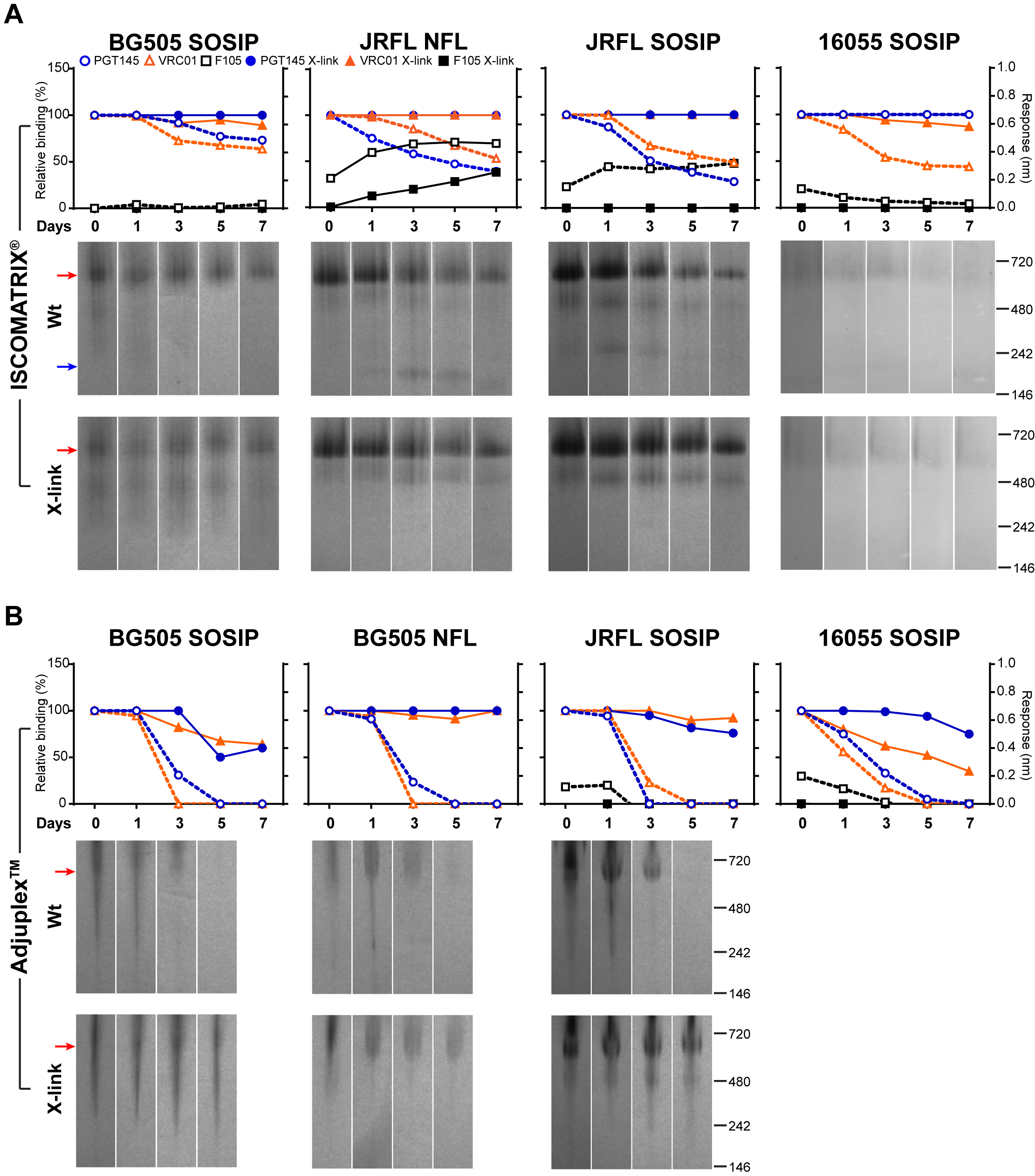

Supplement: S2 Fig — Trimers formulated in ISCOMATRIX adjuvant (A) or Adjuplex (B) were maintained at 37°C and assessed for binding to VRC01, PGT145 and F105 by BLI and stability visualized by BN-PAGE (red arrow, trimer; blue arrow, monomer). The experiments were performed two times independently. (TIF) [file ppat.1005767.s002.tif]

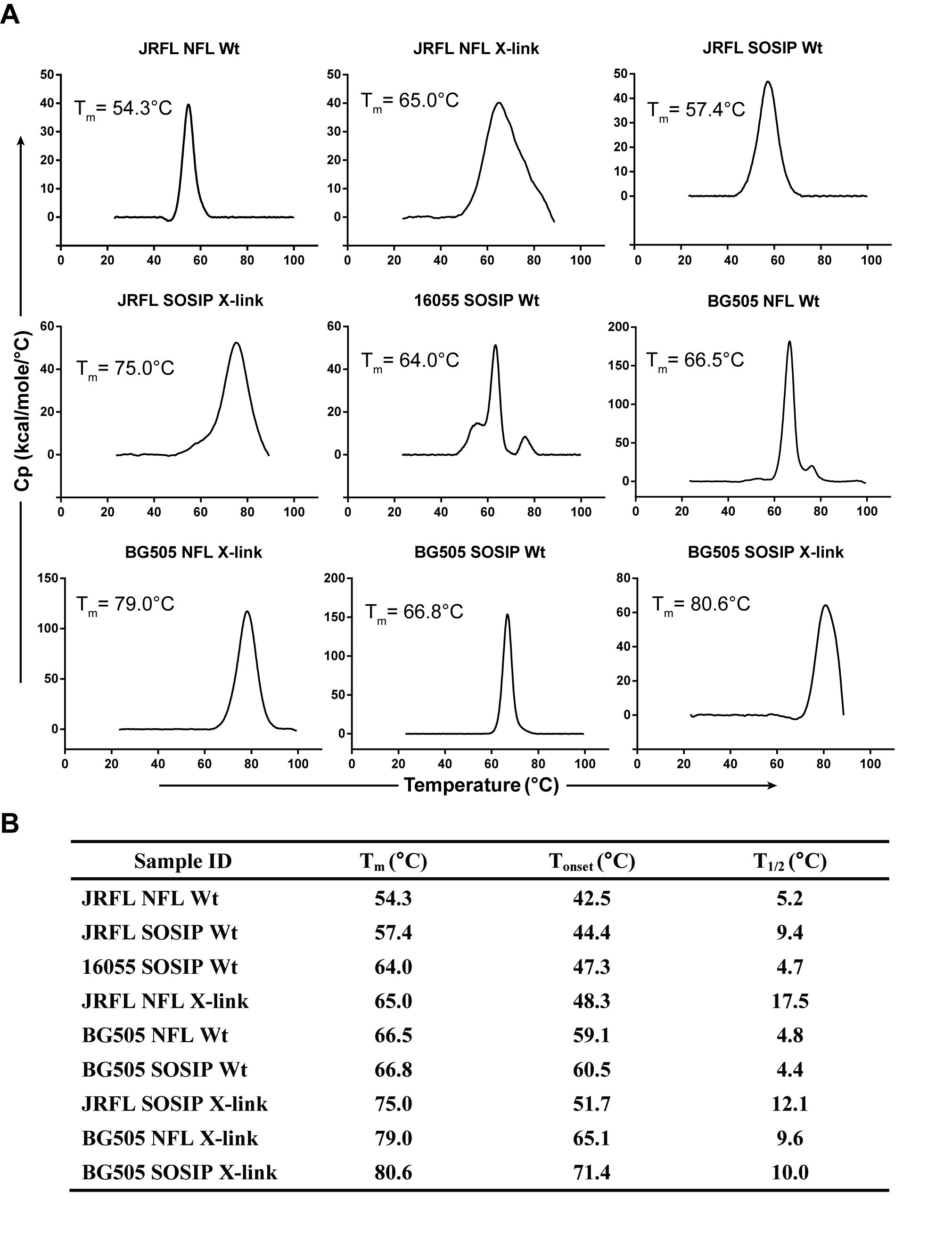

Supplement: S3 Fig — (A) Thermal transition melting (Tm) curves of the different trimers with corresponding values (B) are shown (see Methods). All data are representative of at least two independent experiments. (TIF) [file ppat.1005767.s003.tif]

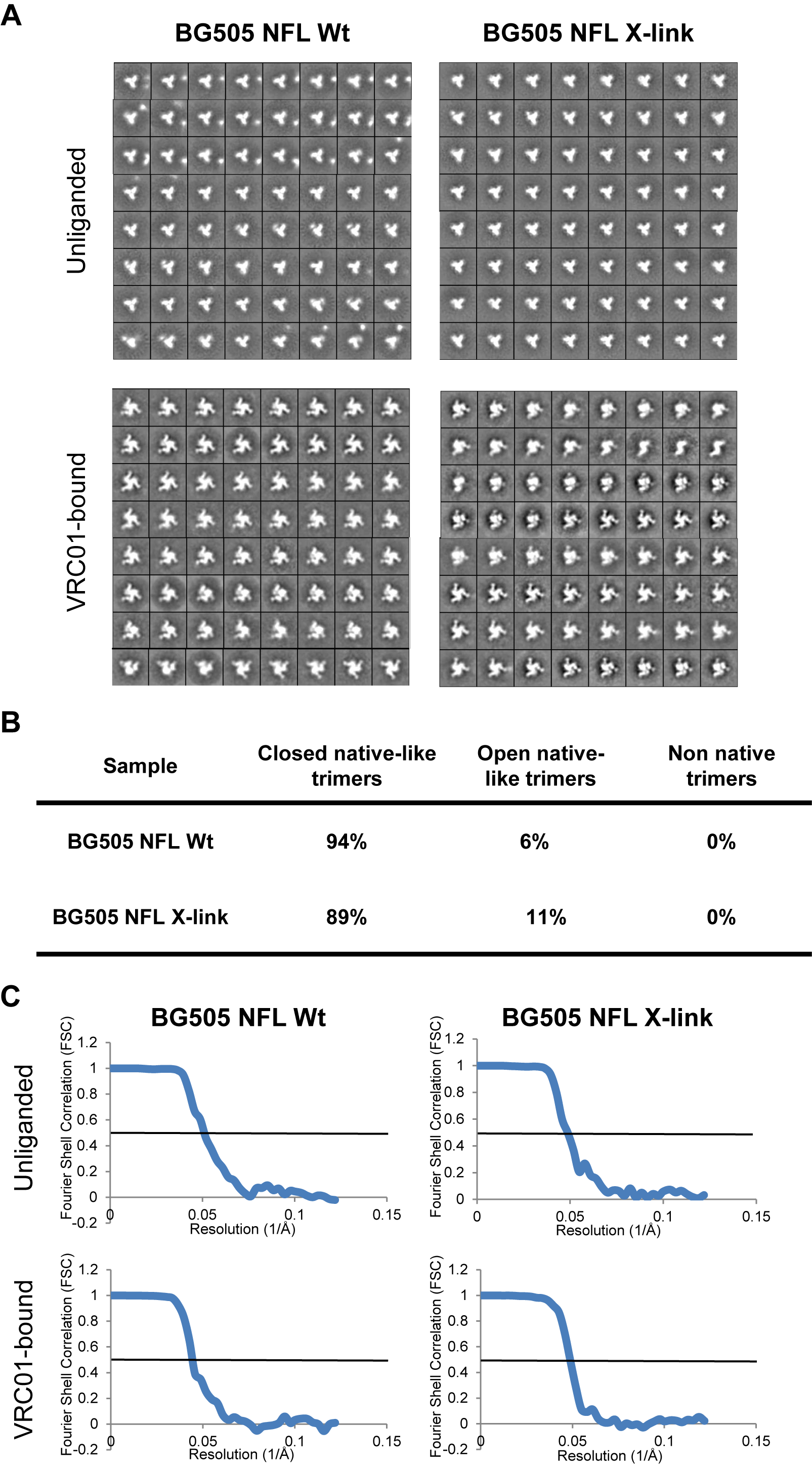

Supplement: S4 Fig — (A) 2D class averages of Wt (left) and X-link BG505 NFL trimers in the unliganded (top) or VRC01-bound state (bottom). (B) Distribution of native closed, native open or non-native like trimers of the unliganded Wt or X-link BG505 NFL samples were determined by analysis of over 2000 individual particles (see Methods). (C) The resolutions of the EM reconstructions were calculated from the Fourier Shell Correlation (FSC) using a cut-off of 0.5. The final resolutions obtained for the different BG505 NFL trimers obtained are as follows: unliganded Wt, 19 Å (upper left, EMD-8270); unliganded X-link, 20 Å (upper right, EMD-8271); VRC01-bound Wt, 21 Å (lower left, EMD-8269); VRC01-bound X-link, 20 Å (lower right, EMD-8268). (TIF) [file ppat.1005767.s004.tif]

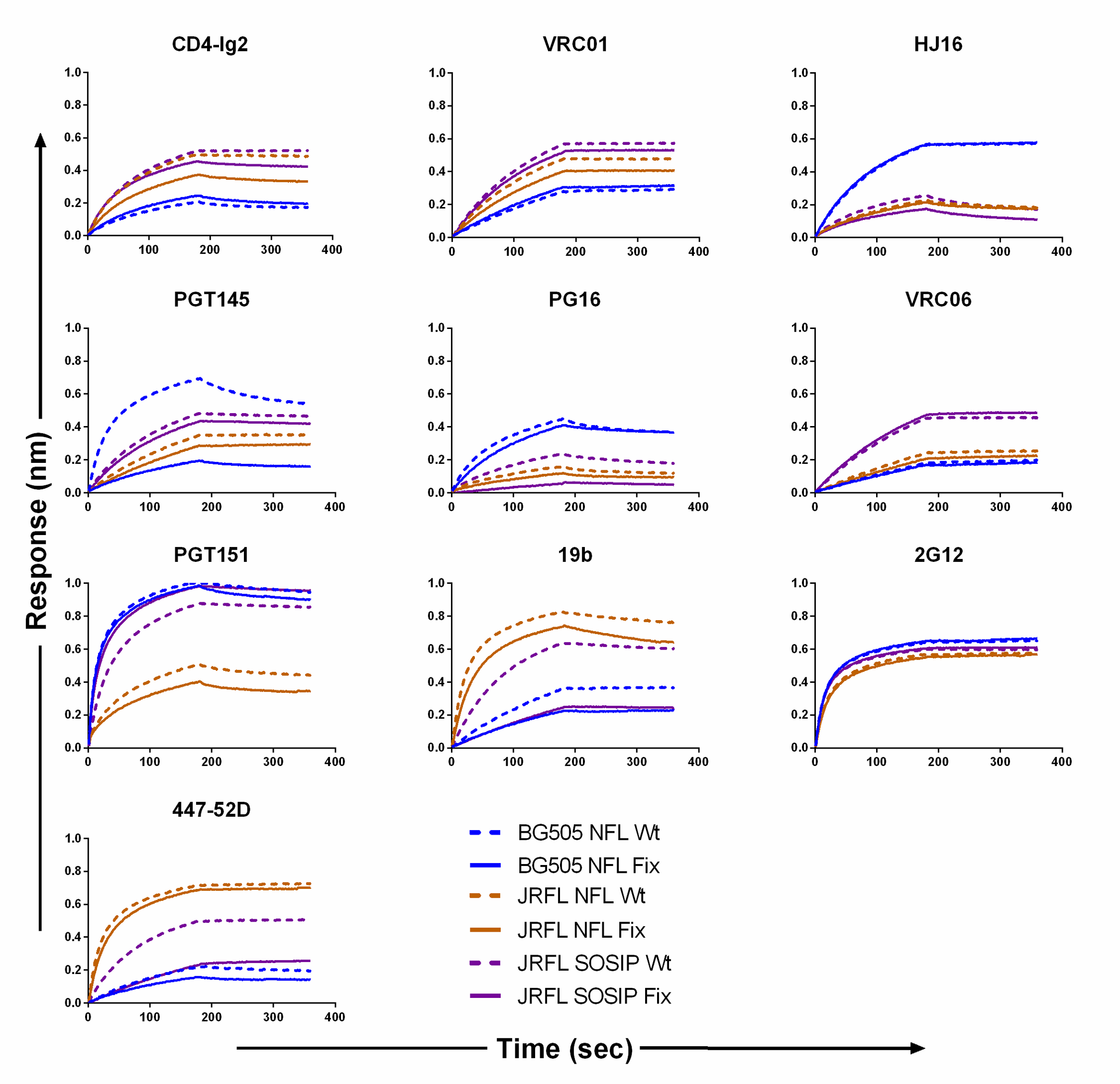

Supplement: S5 Fig — Antibody binding profiles of Wt (dashed lines) or X-link (solid lines) BG505 (blue) and JRFL (red) NFL were assessed by BLI using anti-human Fc sensors to capture the mAb with the trimer in solution. The different classes of mAbs tested include: CD4 binding site directed bNAbs (CD4-Ig2, VRC01, HJ16), trimer preferring bNAbs (PGT145, PG16, VRC06, PGT151), glycan dependent bNAb 2G12, and V3 directed mAbs (447-52D and 19b). All data are representative of at least two independent experiments. (TIF) [file ppat.1005767.s005.tif]

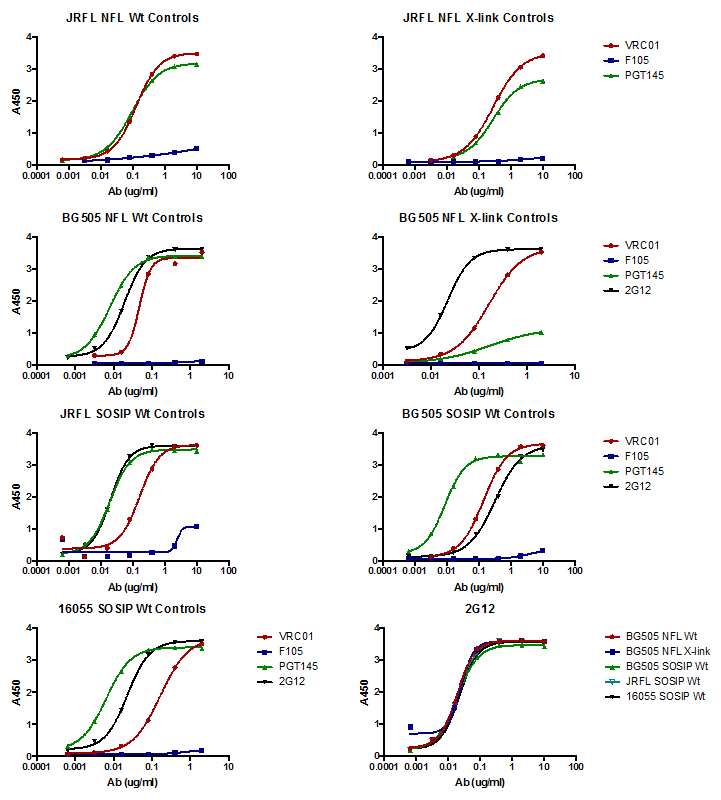

Supplement: S6 Fig — VRC01, F105 and PGT145 binding levels were used to determine the quality of each trimer immunogen captured by His6 C-terminal tag on the ELISA plates. 2G12 was used to confirm that the same amount of trimer was captured in each well of the ELISA plate. (TIF) [file ppat.1005767.s006.tif]

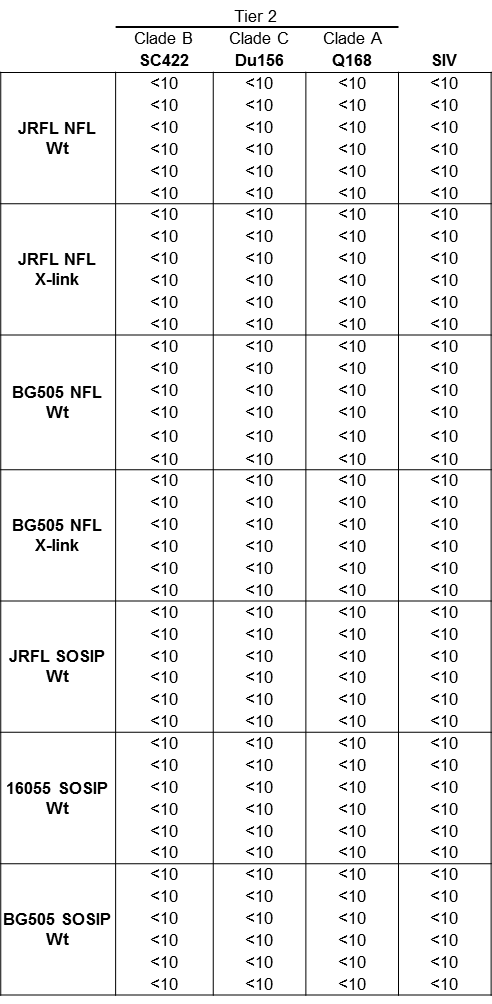

Supplement: S7 Fig — Neutralization capacity of week 26 sera from guinea pigs (n = 6) immunized with select trimers (listed on the left) at 0, 4, 12, 24 weeks against a panel of heterologous tier 2 viruses was determined by the TZM-bl neutralization assay. ID50 values are shown. No breadth was observed from the limited tier 2 panel assessed. SIV was used as a negative control. (TIF) [file ppat.1005767.s007.tif]

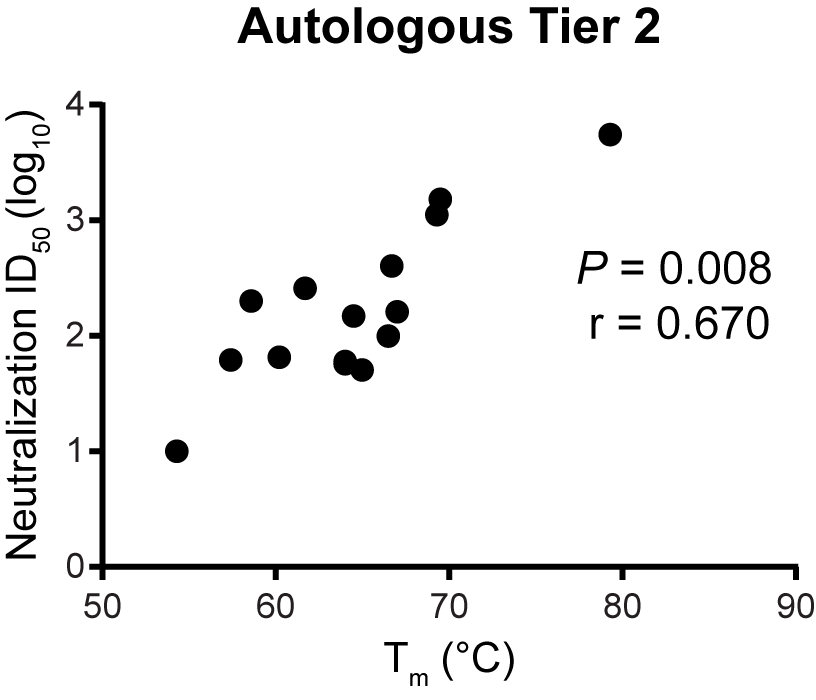

Supplement: S8 Fig — The autologous neutralization ID50 and trimer Tm values from the rabbit study reported in de Taeye et al. (Cell, 2015) were plotted together with our data as presented in Fig 7B. The Tm values are as follows: our study (JRFL NFL Wt, 54.3°C; JRFL SOSIP Wt, 57.4°C; 16055 SOSIP Wt, 64.0°C; JRFL NFL X-link, 65.0°C; BG505 NFL Wt, 66.5°C; BG505 SOSIP Wt, 67.0°C; BG505 NFL X-link, 79.3°C) and de Taeye’s study (B41 SOSIP Wt, 58.6°C; AMC008 SOSIP Wt, 60.2°C; B41 SOSIP v4.1, 61.7°C; AMC008 SOSIP v4.2, 64.0°C; AMC008 SOSIP v4.1, 64.5°C; BG505 SOSIP Wt, 66.7°C; BG505 SOSIP v4.2, 69.3°C; BG505 SOSIP v4.1, 69.5°C). Spearman’s rank correlation analysis showed a statistically significant correlation between the autologous neutralization ID50 and trimer Tm, P value is 0.008 and r value is 0.670. (TIF) [file ppat.1005767.s008.tif]

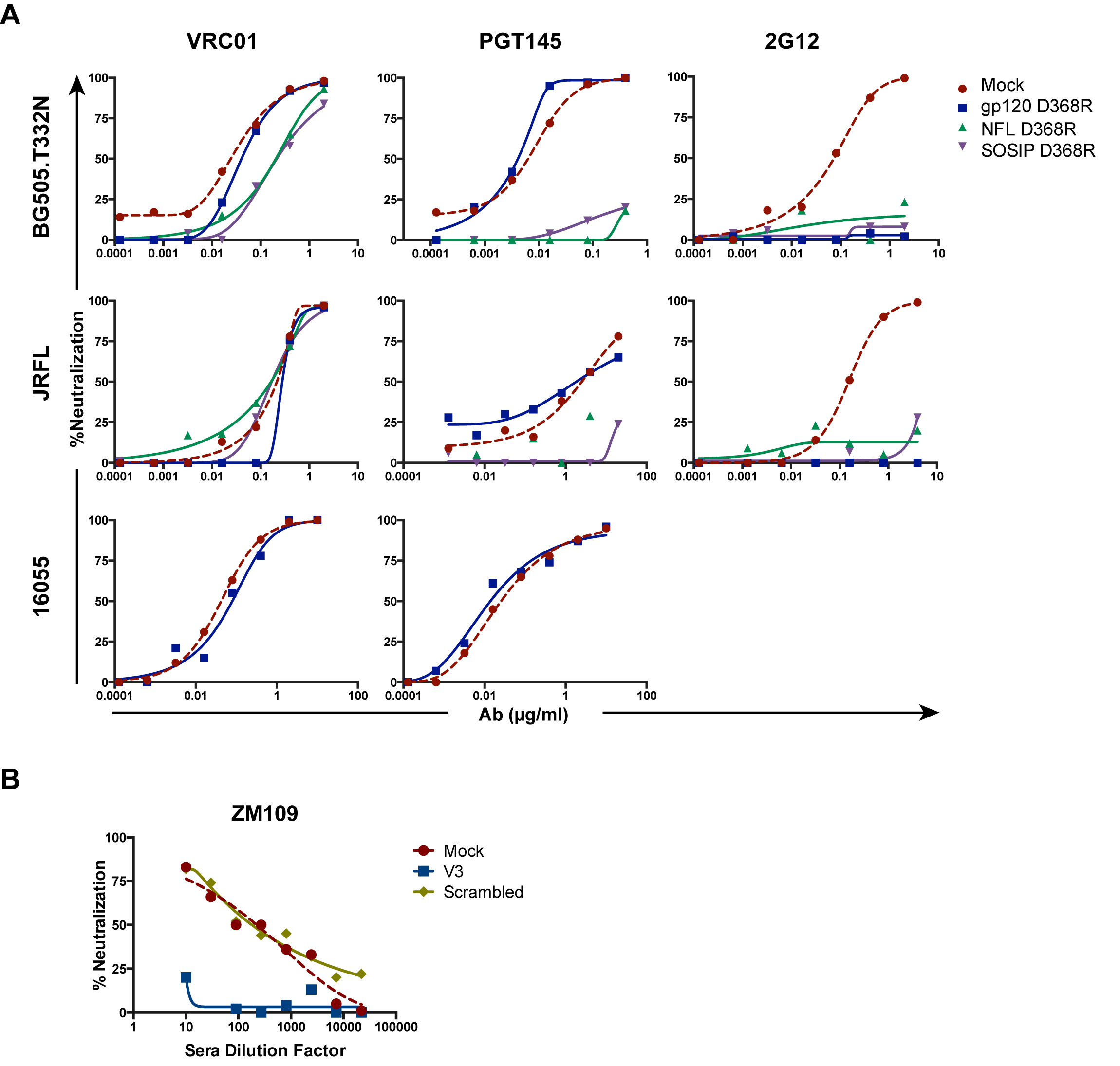

Supplement: S9 Fig — (A) Strain matched CD4bs “knockout” gp120 D368R monomer, NFL/SOSIP D368R trimers were preincubated with mAbs (listed above) prior to addition of pseudovirus (listed on the left) in the TZM-bl neutralization assay. VRC01 (CD4bs-directed) and 2G12 (glycan reactive) can bind monomer and trimer while PGT145 binds trimer. (B) Representative example of V3 peptide inhibition assay. Sera samples were preincubated with media (mock), V3 peptide, or scrambled peptide prior to addition of pseudovirus in the TZM-bl neutralization assay. (TIF) [file ppat.1005767.s009.tif]

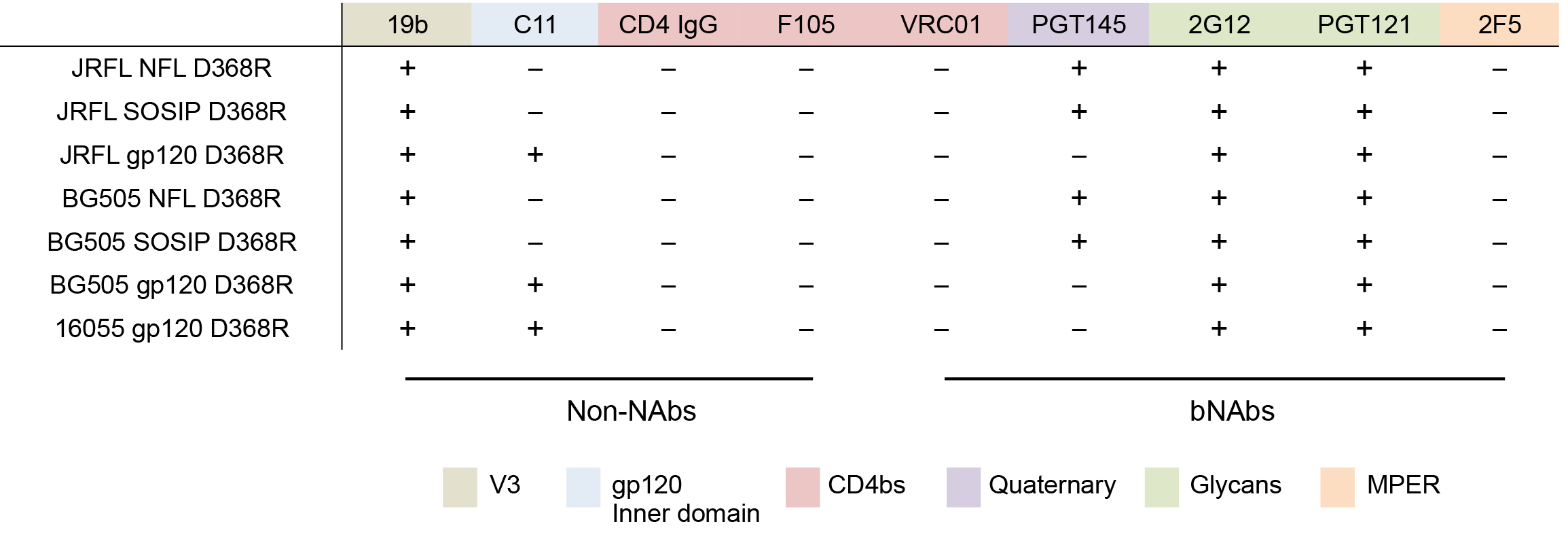

Supplement: S10 Fig — Antibody binding profiles of the D368R variants used in the sera mapping experiment were assessed by BLI using anti-human Fc sensors to capture the mAbs with the trimer variants in solution. Results are summarized in the figure with “+” for binding and “-” for no binding. (TIF) [file ppat.1005767.s010.tif]
